# Search for third-generation scalar leptoquarks decaying to a top quark and a $\tau$ lepton at $\sqrt{s}=13\,\text {Te}\text {V}$

**DOI:** 10.1140/epjc/s10052-018-6143-z

**Published:** 2018-09-03

**Authors:** A. M. Sirunyan, A. Tumasyan, W. Adam, F. Ambrogi, E. Asilar, T. Bergauer, J. Brandstetter, E. Brondolin, M. Dragicevic, J. Erö, A. Escalante Del Valle, M. Flechl, M. Friedl, R. Frühwirth, V. M. Ghete, J. Grossmann, J. Hrubec, M. Jeitler, A. König, N. Krammer, I. Krätschmer, D. Liko, T. Madlener, I. Mikulec, E. Pree, N. Rad, H. Rohringer, J. Schieck, R. Schöfbeck, M. Spanring, D. Spitzbart, A. Taurok, W. Waltenberger, J. Wittmann, C.-E. Wulz, M. Zarucki, V. Chekhovsky, V. Mossolov, J. Suarez Gonzalez, E. A. De Wolf, D. Di Croce, X. Janssen, J. Lauwers, M. Pieters, M. Van De Klundert, H. Van Haevermaet, P. Van Mechelen, N. Van Remortel, S. Abu Zeid, F. Blekman, J. D’Hondt, I. De Bruyn, J. De Clercq, K. Deroover, G. Flouris, D. Lontkovskyi, S. Lowette, I. Marchesini, S. Moortgat, L. Moreels, Q. Python, K. Skovpen, S. Tavernier, W. Van Doninck, P. Van Mulders, I. Van Parijs, D. Beghin, B. Bilin, H. Brun, B. Clerbaux, G. De Lentdecker, H. Delannoy, B. Dorney, G. Fasanella, L. Favart, R. Goldouzian, A. Grebenyuk, A. K. Kalsi, T. Lenzi, J. Luetic, T. Maerschalk, T. Seva, E. Starling, C. Vander Velde, P. Vanlaer, D. Vannerom, R. Yonamine, F. Zenoni, T. Cornelis, D. Dobur, A. Fagot, M. Gul, I. Khvastunov, D. Poyraz, C. Roskas, D. Trocino, M. Tytgat, W. Verbeke, M. Vit, N. Zaganidis, H. Bakhshiansohi, O. Bondu, S. Brochet, G. Bruno, C. Caputo, A. Caudron, P. David, S. De Visscher, C. Delaere, M. Delcourt, B. Francois, A. Giammanco, G. Krintiras, V. Lemaitre, A. Magitteri, A. Mertens, M. Musich, K. Piotrzkowski, L. Quertenmont, A. Saggio, M. Vidal Marono, S. Wertz, J. Zobec, W. L. Aldá Júnior, F. L. Alves, G. A. Alves, L. Brito, G. Correia Silva, C. Hensel, A. Moraes, M. E. Pol, P. Rebello Teles, E. Belchior Batista Das Chagas, W. Carvalho, J. Chinellato, E. Coelho, E. M. Da Costa, G. G. Da Silveira, D. De Jesus Damiao, S. Fonseca De Souza, L. M. Huertas Guativa, H. Malbouisson, M. Medina Jaime, M. Melo De Almeida, C. Mora Herrera, L. Mundim, H. Nogima, L. J. Sanchez Rosas, A. Santoro, A. Sznajder, M. Thiel, E. J. Tonelli Manganote, F. Torres Da Silva De Araujo, A. Vilela Pereira, S. Ahuja, C. A. Bernardes, T. R. Fernandez Perez Tomei, E. M. Gregores, P. G. Mercadante, S. F. Novaes, Sandra S. Padula, D. Romero Abad, J. C. Ruiz Vargas, A. Aleksandrov, R. Hadjiiska, P. Iaydjiev, A. Marinov, M. Misheva, M. Rodozov, M. Shopova, G. Sultanov, A. Dimitrov, L. Litov, B. Pavlov, P. Petkov, W. Fang, X. Gao, L. Yuan, M. Ahmad, J. G. Bian, G. M. Chen, H. S. Chen, M. Chen, Y. Chen, C. H. Jiang, D. Leggat, H. Liao, Z. Liu, F. Romeo, S. M. Shaheen, A. Spiezia, J. Tao, C. Wang, Z. Wang, E. Yazgan, H. Zhang, J. Zhao, Y. Ban, G. Chen, J. Li, Q. Li, S. Liu, Y. Mao, S. J. Qian, D. Wang, Z. Xu, Y. Wang, C. Avila, A. Cabrera, C. A. Carrillo Montoya, L. F. Chaparro Sierra, C. Florez, C. F. González Hernández, J. D. Ruiz Alvarez, M. A. Segura Delgado, B. Courbon, N. Godinovic, D. Lelas, I. Puljak, P. M. Ribeiro Cipriano, T. Sculac, Z. Antunovic, M. Kovac, V. Brigljevic, D. Ferencek, K. Kadija, B. Mesic, A. Starodumov, T. Susa, M. W. Ather, A. Attikis, G. Mavromanolakis, J. Mousa, C. Nicolaou, F. Ptochos, P. A. Razis, H. Rykaczewski, M. Finger, M. Finger, E. Carrera Jarrin, A. A. Abdelalim, S. Elgammal, A. Ellithi Kamel, S. Bhowmik, R. K. Dewanjee, M. Kadastik, L. Perrini, M. Raidal, C. Veelken, P. Eerola, H. Kirschenmann, J. Pekkanen, M. Voutilainen, J. Havukainen, J. K. Heikkilä, T. Järvinen, V. Karimäki, R. Kinnunen, T. Lampén, K. Lassila-Perini, S. Laurila, S. Lehti, T. Lindén, P. Luukka, T. Mäenpää, H. Siikonen, E. Tuominen, J. Tuominiemi, T. Tuuva, M. Besancon, F. Couderc, M. Dejardin, D. Denegri, J. L. Faure, F. Ferri, S. Ganjour, S. Ghosh, A. Givernaud, P. Gras, G. Hamel de Monchenault, P. Jarry, C. Leloup, E. Locci, M. Machet, J. Malcles, G. Negro, J. Rander, A. Rosowsky, M. Ö. Sahin, M. Titov, A. Abdulsalam, C. Amendola, I. Antropov, S. Baffioni, F. Beaudette, P. Busson, L. Cadamuro, C. Charlot, R. Granier de Cassagnac, M. Jo, I. Kucher, S. Lisniak, A. Lobanov, J. Martin Blanco, M. Nguyen, C. Ochando, G. Ortona, P. Paganini, P. Pigard, R. Salerno, J. B. Sauvan, Y. Sirois, A. G. Stahl Leiton, Y. Yilmaz, A. Zabi, A. Zghiche, J.-L. Agram, J. Andrea, D. Bloch, J.-M. Brom, M. Buttignol, E. C. Chabert, C. Collard, E. Conte, X. Coubez, F. Drouhin, J.-C. Fontaine, D. Gelé, U. Goerlach, M. Jansová, P. Juillot, A.-C. Le Bihan, N. Tonon, P. Van Hove, S. Gadrat, S. Beauceron, C. Bernet, G. Boudoul, N. Chanon, R. Chierici, D. Contardo, P. Depasse, H. El Mamouni, J. Fay, L. Finco, S. Gascon, M. Gouzevitch, G. Grenier, B. Ille, F. Lagarde, I. B. Laktineh, H. Lattaud, M. Lethuillier, L. Mirabito, A. L. Pequegnot, S. Perries, A. Popov, V. Sordini, M. Vander Donckt, S. Viret, S. Zhang, T. Toriashvili, Z. Tsamalaidze, C. Autermann, L. Feld, M. K. Kiesel, K. Klein, M. Lipinski, M. Preuten, C. Schomakers, J. Schulz, M. Teroerde, B. Wittmer, V. Zhukov, A. Albert, D. Duchardt, M. Endres, M. Erdmann, S. Erdweg, T. Esch, R. Fischer, A. Güth, T. Hebbeker, C. Heidemann, K. Hoepfner, S. Knutzen, M. Merschmeyer, A. Meyer, P. Millet, S. Mukherjee, T. Pook, M. Radziej, H. Reithler, M. Rieger, F. Scheuch, D. Teyssier, S. Thüer, G. Flügge, B. Kargoll, T. Kress, A. Künsken, T. Müller, A. Nehrkorn, A. Nowack, C. Pistone, O. Pooth, A. Stahl, M. Aldaya Martin, T. Arndt, C. Asawatangtrakuldee, K. Beernaert, O. Behnke, U. Behrens, A. Bermúdez Martínez, A. A. Bin Anuar, K. Borras, V. Botta, A. Campbell, P. Connor, C. Contreras-Campana, F. Costanza, A. De Wit, C. Diez Pardos, G. Eckerlin, D. Eckstein, T. Eichhorn, E. Eren, E. Gallo, J. Garay Garcia, A. Geiser, J. M. Grados Luyando, A. Grohsjean, P. Gunnellini, M. Guthoff, A. Harb, J. Hauk, M. Hempel, H. Jung, M. Kasemann, J. Keaveney, C. Kleinwort, I. Korol, D. Krücker, W. Lange, A. Lelek, T. Lenz, K. Lipka, W. Lohmann, R. Mankel, I.-A. Melzer-Pellmann, A. B. Meyer, M. Meyer, M. Missiroli, G. Mittag, J. Mnich, A. Mussgiller, D. Pitzl, A. Raspereza, M. Savitskyi, P. Saxena, R. Shevchenko, N. Stefaniuk, H. Tholen, G. P. Van Onsem, R. Walsh, Y. Wen, K. Wichmann, C. Wissing, O. Zenaiev, R. Aggleton, S. Bein, V. Blobel, M. Centis Vignali, T. Dreyer, E. Garutti, D. Gonzalez, J. Haller, A. Hinzmann, M. Hoffmann, A. Karavdina, G. Kasieczka, R. Klanner, R. Kogler, N. Kovalchuk, S. Kurz, D. Marconi, J. Multhaup, M. Niedziela, D. Nowatschin, T. Peiffer, A. Perieanu, A. Reimers, C. Scharf, P. Schleper, A. Schmidt, S. Schumann, J. Schwandt, J. Sonneveld, H. Stadie, G. Steinbrück, F. M. Stober, M. Stöver, D. Troendle, E. Usai, A. Vanhoefer, B. Vormwald, M. Akbiyik, C. Barth, M. Baselga, S. Baur, E. Butz, R. Caspart, T. Chwalek, F. Colombo, W. De Boer, A. Dierlamm, N. Faltermann, B. Freund, R. Friese, M. Giffels, M. A. Harrendorf, F. Hartmann, S. M. Heindl, U. Husemann, F. Kassel, S. Kudella, H. Mildner, M. U. Mozer, Th. Müller, M. Plagge, G. Quast, K. Rabbertz, M. Schröder, I. Shvetsov, G. Sieber, H. J. Simonis, R. Ulrich, S. Wayand, M. Weber, T. Weiler, S. Williamson, C. Wöhrmann, R. Wolf, G. Anagnostou, G. Daskalakis, T. Geralis, A. Kyriakis, D. Loukas, I. Topsis-Giotis, G. Karathanasis, S. Kesisoglou, A. Panagiotou, N. Saoulidou, E. Tziaferi, K. Kousouris, I. Papakrivopoulos, I. Evangelou, C. Foudas, P. Gianneios, P. Katsoulis, P. Kokkas, S. Mallios, N. Manthos, I. Papadopoulos, E. Paradas, J. Strologas, F. A. Triantis, D. Tsitsonis, M. Csanad, N. Filipovic, G. Pasztor, O. Surányi, G. I. Veres, G. Bencze, C. Hajdu, D. Horvath, Á. Hunyadi, F. Sikler, T. Á. Vámi, V. Veszpremi, G. Vesztergombi, N. Beni, S. Czellar, J. Karancsi, A. Makovec, J. Molnar, Z. Szillasi, M. Bartók, P. Raics, Z. L. Trocsanyi, B. Ujvari, S. Choudhury, J. R. Komaragiri, S. Bahinipati, P. Mal, K. Mandal, A. Nayak, D. K. Sahoo, N. Sahoo, S. K. Swain, S. Bansal, S. B. Beri, V. Bhatnagar, R. Chawla, N. Dhingra, R. Gupta, A. Kaur, M. Kaur, S. Kaur, R. Kumar, P. Kumari, A. Mehta, S. Sharma, J. B. Singh, G. Walia, A. Bhardwaj, S. Chauhan, B. C. Choudhary, R. B. Garg, S. Keshri, A. Kumar, Ashok Kumar, S. Malhotra, M. Naimuddin, K. Ranjan, Aashaq Shah, R. Sharma, R. Bhardwaj, R. Bhattacharya, S. Bhattacharya, U. Bhawandeep, D. Bhowmik, S. Dey, S. Dutt, S. Dutta, S. Ghosh, N. Majumdar, A. Modak, K. Mondal, S. Mukhopadhyay, S. Nandan, A. Purohit, P. K. Rout, A. Roy, S. Roy Chowdhury, S. Sarkar, M. Sharan, B. Singh, S. Thakur, P. K. Behera, R. Chudasama, D. Dutta, V. Jha, V. Kumar, A. K. Mohanty, P. K. Netrakanti, L. M. Pant, P. Shukla, A. Topkar, T. Aziz, S. Dugad, B. Mahakud, S. Mitra, G. B. Mohanty, N. Sur, B. Sutar, S. Banerjee, S. Bhattacharya, S. Chatterjee, P. Das, M. Guchait, Sa. Jain, S. Kumar, M. Maity, G. Majumder, K. Mazumdar, T. Sarkar, N. Wickramage, S. Chauhan, S. Dube, V. Hegde, A. Kapoor, K. Kothekar, S. Pandey, A. Rane, S. Sharma, S. Chenarani, E. Eskandari Tadavani, S. M. Etesami, M. Khakzad, M. Mohammadi Najafabadi, M. Naseri, S. Paktinat Mehdiabadi, F. Rezaei Hosseinabadi, B. Safarzadeh, M. Zeinali, M. Felcini, M. Grunewald, M. Abbrescia, C. Calabria, A. Colaleo, D. Creanza, L. Cristella, N. De Filippis, M. De Palma, A. Di Florio, F. Errico, L. Fiore, G. Iaselli, S. Lezki, G. Maggi, M. Maggi, B. Marangelli, G. Miniello, S. My, S. Nuzzo, A. Pompili, G. Pugliese, R. Radogna, A. Ranieri, G. Selvaggi, A. Sharma, L. Silvestris, R. Venditti, P. Verwilligen, G. Zito, G. Abbiendi, C. Battilana, D. Bonacorsi, L. Borgonovi, S. Braibant-Giacomelli, R. Campanini, P. Capiluppi, A. Castro, F. R. Cavallo, S. S. Chhibra, G. Codispoti, M. Cuffiani, G. M. Dallavalle, F. Fabbri, A. Fanfani, D. Fasanella, P. Giacomelli, C. Grandi, L. Guiducci, F. Iemmi, S. Marcellini, G. Masetti, A. Montanari, F. L. Navarria, A. Perrotta, A. M. Rossi, T. Rovelli, G. P. Siroli, N. Tosi, S. Albergo, S. Costa, A. Di Mattia, F. Giordano, R. Potenza, A. Tricomi, C. Tuve, G. Barbagli, K. Chatterjee, V. Ciulli, C. Civinini, R. D’Alessandro, E. Focardi, G. Latino, P. Lenzi, M. Meschini, S. Paoletti, L. Russo, G. Sguazzoni, D. Strom, L. Viliani, L. Benussi, S. Bianco, F. Fabbri, D. Piccolo, F. Primavera, V. Calvelli, F. Ferro, F. Ravera, E. Robutti, S. Tosi, A. Benaglia, A. Beschi, L. Brianza, F. Brivio, V. Ciriolo, M. E. Dinardo, S. Fiorendi, S. Gennai, A. Ghezzi, P. Govoni, M. Malberti, S. Malvezzi, R. A. Manzoni, D. Menasce, L. Moroni, M. Paganoni, K. Pauwels, D. Pedrini, S. Pigazzini, S. Ragazzi, T. Tabarelli de Fatis, S. Buontempo, N. Cavallo, S. Di Guida, F. Fabozzi, F. Fienga, A. O. M. Iorio, W. A. Khan, L. Lista, S. Meola, P. Paolucci, C. Sciacca, F. Thyssen, P. Azzi, N. Bacchetta, L. Benato, D. Bisello, A. Boletti, R. Carlin, A. Carvalho Antunes De Oliveira, P. Checchia, M. Dall’Osso, P. De Castro Manzano, T. Dorigo, F. Gasparini, U. Gasparini, A. Gozzelino, S. Lacaprara, P. Lujan, M. Margoni, A. T. Meneguzzo, N. Pozzobon, P. Ronchese, R. Rossin, F. Simonetto, A. Tiko, E. Torassa, M. Zanetti, P. Zotto, G. Zumerle, A. Braghieri, A. Magnani, P. Montagna, S. P. Ratti, V. Re, M. Ressegotti, C. Riccardi, P. Salvini, I. Vai, P. Vitulo, L. Alunni Solestizi, M. Biasini, G. M. Bilei, C. Cecchi, D. Ciangottini, L. Fanò, P. Lariccia, R. Leonardi, E. Manoni, G. Mantovani, V. Mariani, M. Menichelli, A. Rossi, A. Santocchia, D. Spiga, K. Androsov, P. Azzurri, G. Bagliesi, L. Bianchini, T. Boccali, L. Borrello, R. Castaldi, M. A. Ciocci, R. Dell’Orso, G. Fedi, L. Giannini, A. Giassi, M. T. Grippo, F. Ligabue, T. Lomtadze, E. Manca, G. Mandorli, A. Messineo, F. Palla, A. Rizzi, P. Spagnolo, R. Tenchini, G. Tonelli, A. Venturi, P. G. Verdini, L. Barone, F. Cavallari, M. Cipriani, N. Daci, D. Del Re, E. Di Marco, M. Diemoz, S. Gelli, E. Longo, F. Margaroli, B. Marzocchi, P. Meridiani, G. Organtini, R. Paramatti, F. Preiato, S. Rahatlou, C. Rovelli, F. Santanastasio, N. Amapane, R. Arcidiacono, S. Argiro, M. Arneodo, N. Bartosik, R. Bellan, C. Biino, N. Cartiglia, R. Castello, F. Cenna, M. Costa, R. Covarelli, A. Degano, N. Demaria, B. Kiani, C. Mariotti, S. Maselli, E. Migliore, V. Monaco, E. Monteil, M. Monteno, M. M. Obertino, L. Pacher, N. Pastrone, M. Pelliccioni, G. L. Pinna Angioni, A. Romero, M. Ruspa, R. Sacchi, K. Shchelina, V. Sola, A. Solano, A. Staiano, P. Traczyk, S. Belforte, M. Casarsa, F. Cossutti, G. Della Ricca, A. Zanetti, D. H. Kim, G. N. Kim, M. S. Kim, J. Lee, S. Lee, S. W. Lee, C. S. Moon, Y. D. Oh, S. Sekmen, D. C. Son, Y. C. Yang, H. Kim, D. H. Moon, G. Oh, J. A. Brochero Cifuentes, J. Goh, T. J. Kim, S. Cho, S. Choi, Y. Go, D. Gyun, S. Ha, B. Hong, Y. Jo, Y. Kim, K. Lee, K. S. Lee, S. Lee, J. Lim, S. K. Park, Y. Roh, J. Almond, J. Kim, J. S. Kim, H. Lee, K. Lee, K. Nam, S. B. Oh, B. C. Radburn-Smith, S. h. Seo, U. K. Yang, H. D. Yoo, G. B. Yu, H. Kim, J. H. Kim, J. S. H. Lee, I. C. Park, Y. Choi, C. Hwang, J. Lee, I. Yu, V. Dudenas, A. Juodagalvis, J. Vaitkus, I. Ahmed, Z. A. Ibrahim, M. A. B. Md Ali, F. Mohamad Idris, W. A. T. Wan Abdullah, M. N. Yusli, Z. Zolkapli, M. C. Duran-Osuna, H. Castilla-Valdez, E. De La Cruz-Burelo, G. Ramirez-Sanchez, I. Heredia-De La Cruz, R. I. Rabadan-Trejo, R. Lopez-Fernandez, J. Mejia Guisao, R Reyes-Almanza, A. Sanchez-Hernandez, S. Carrillo Moreno, C. Oropeza Barrera, F. Vazquez Valencia, J. Eysermans, I. Pedraza, H. A. Salazar Ibarguen, C. Uribe Estrada, A. Morelos Pineda, D. Krofcheck, P. H. Butler, A. Ahmad, M. Ahmad, Q. Hassan, H. R. Hoorani, A. Saddique, M. A. Shah, M. Shoaib, M. Waqas, H. Bialkowska, M. Bluj, B. Boimska, T. Frueboes, M. Górski, M. Kazana, K. Nawrocki, M. Szleper, P. Zalewski, K. Bunkowski, A. Byszuk, K. Doroba, A. Kalinowski, M. Konecki, J. Krolikowski, M. Misiura, M. Olszewski, A. Pyskir, M. Walczak, P. Bargassa, C. Beirão Da Cruz E Silva, A. Di Francesco, P. Faccioli, B. Galinhas, M. Gallinaro, J. Hollar, N. Leonardo, L. Lloret Iglesias, M. V. Nemallapudi, J. Seixas, G. Strong, O. Toldaiev, D. Vadruccio, J. Varela, S. Afanasiev, P. Bunin, M. Gavrilenko, I. Golutvin, I. Gorbunov, A. Kamenev, V. Karjavin, A. Lanev, A. Malakhov, V. Matveev, P. Moisenz, V. Palichik, V. Perelygin, S. Shmatov, S. Shulha, N. Skatchkov, V. Smirnov, N. Voytishin, A. Zarubin, Y. Ivanov, V. Kim, E. Kuznetsova, P. Levchenko, V. Murzin, V. Oreshkin, I. Smirnov, D. Sosnov, V. Sulimov, L. Uvarov, S. Vavilov, A. Vorobyev, Yu. Andreev, A. Dermenev, S. Gninenko, N. Golubev, A. Karneyeu, M. Kirsanov, N. Krasnikov, A. Pashenkov, D. Tlisov, A. Toropin, V. Epshteyn, V. Gavrilov, N. Lychkovskaya, V. Popov, I. Pozdnyakov, G. Safronov, A. Spiridonov, A. Stepennov, V. Stolin, M. Toms, E. Vlasov, A. Zhokin, T. Aushev, A. Bylinkin, M. Chadeeva, P. Parygin, D. Philippov, S. Polikarpov, E. Popova, V. Rusinov, V. Andreev, M. Azarkin, I. Dremin, M. Kirakosyan, S. V. Rusakov, A. Terkulov, A. Baskakov, A. Belyaev, E. Boos, V. Bunichev, M. Dubinin, L. Dudko, A. Ershov, A. Gribushin, V. Klyukhin, O. Kodolova, I. Lokhtin, I. Miagkov, S. Obraztsov, M. Perfilov, V. Savrin, V. Blinov, D. Shtol, Y. Skovpen, I. Azhgirey, I. Bayshev, S. Bitioukov, D. Elumakhov, A. Godizov, V. Kachanov, A. Kalinin, D. Konstantinov, P. Mandrik, V. Petrov, R. Ryutin, A. Sobol, S. Troshin, N. Tyurin, A. Uzunian, A. Volkov, A. Babaev, P. Adzic, P. Cirkovic, D. Devetak, M. Dordevic, J. Milosevic, J. Alcaraz Maestre, A. Álvarez Fernández, I. Bachiller, M. Barrio Luna, M. Cerrada, N. Colino, B. De La Cruz, A. Delgado Peris, C. Fernandez Bedoya, J. P. Fernández Ramos, J. Flix, M. C. Fouz, O. Gonzalez Lopez, S. Goy Lopez, J. M. Hernandez, M. I. Josa, D. Moran, A. Pérez-Calero Yzquierdo, J. Puerta Pelayo, I. Redondo, L. Romero, M. S. Soares, A. Triossi, C. Albajar, J. F. de Trocóniz, J. Cuevas, C. Erice, J. Fernandez Menendez, S. Folgueras, I. Gonzalez Caballero, J. R. González Fernández, E. Palencia Cortezon, S. Sanchez Cruz, P. Vischia, J. M. Vizan Garcia, I. J. Cabrillo, A. Calderon, B. Chazin Quero, J. Duarte Campderros, M. Fernandez, P. J. Fernández Manteca, A. García Alonso, J. Garcia-Ferrero, G. Gomez, A. Lopez Virto, J. Marco, C. Martinez Rivero, P. Martinez Ruiz del Arbol, F. Matorras, J. Piedra Gomez, C. Prieels, T. Rodrigo, A. Ruiz-Jimeno, L. Scodellaro, N. Trevisani, I. Vila, R. Vilar Cortabitarte, D. Abbaneo, B. Akgun, E. Auffray, P. Baillon, A. H. Ball, D. Barney, J. Bendavid, M. Bianco, A. Bocci, C. Botta, T. Camporesi, M. Cepeda, G. Cerminara, E. Chapon, Y. Chen, D. d’Enterria, A. Dabrowski, V. Daponte, A. David, M. De Gruttola, A. De Roeck, N. Deelen, M. Dobson, T. du Pree, M. Dünser, N. Dupont, A. Elliott-Peisert, P. Everaerts, F. Fallavollita, G. Franzoni, J. Fulcher, W. Funk, D. Gigi, A. Gilbert, K. Gill, F. Glege, D. Gulhan, J. Hegeman, V. Innocente, A. Jafari, P. Janot, O. Karacheban, J. Kieseler, V. Knünz, A. Kornmayer, M. J. Kortelainen, M. Krammer, C. Lange, P. Lecoq, C. Lourenço, M. T. Lucchini, L. Malgeri, M. Mannelli, A. Martelli, F. Meijers, J. A. Merlin, S. Mersi, E. Meschi, P. Milenovic, F. Moortgat, M. Mulders, H. Neugebauer, J. Ngadiuba, S. Orfanelli, L. Orsini, F. Pantaleo, L. Pape, E. Perez, M. Peruzzi, A. Petrilli, G. Petrucciani, A. Pfeiffer, M. Pierini, F. M. Pitters, D. Rabady, A. Racz, T. Reis, G. Rolandi, M. Rovere, H. Sakulin, C. Schäfer, C. Schwick, M. Seidel, M. Selvaggi, A. Sharma, P. Silva, P. Sphicas, A. Stakia, J. Steggemann, M. Stoye, M. Tosi, D. Treille, A. Tsirou, V. Veckalns, M. Verweij, W. D. Zeuner, W. Bertl, L. Caminada, K. Deiters, W. Erdmann, R. Horisberger, Q. Ingram, H. C. Kaestli, D. Kotlinski, U. Langenegger, T. Rohe, S. A. Wiederkehr, M. Backhaus, L. Bäni, P. Berger, B. Casal, G. Dissertori, M. Dittmar, M. Donegà, C. Dorfer, C. Grab, C. Heidegger, D. Hits, J. Hoss, T. Klijnsma, W. Lustermann, M. Marionneau, M. T. Meinhard, D. Meister, F. Micheli, P. Musella, F. Nessi-Tedaldi, F. Pandolfi, J. Pata, F. Pauss, G. Perrin, L. Perrozzi, M. Quittnat, M. Reichmann, D. A. Sanz Becerra, M. Schönenberger, L. Shchutska, V. R. Tavolaro, K. Theofilatos, M. L. Vesterbacka Olsson, R. Wallny, D. H. Zhu, T. K. Aarrestad, C. Amsler, D. Brzhechko, M. F. Canelli, A. De Cosa, R. Del Burgo, S. Donato, C. Galloni, T. Hreus, B. Kilminster, I. Neutelings, D. Pinna, G. Rauco, P. Robmann, D. Salerno, K. Schweiger, C. Seitz, Y. Takahashi, A. Zucchetta, V. Candelise, Y. H. Chang, K. y. Cheng, T. H. Doan, Sh. Jain, R. Khurana, C. M. Kuo, W. Lin, A. Pozdnyakov, S. S. Yu, P. Chang, Y. Chao, K. F. Chen, P. H. Chen, F. Fiori, W.-S. Hou, Y. Hsiung, Arun Kumar, Y. F. Liu, R.-S. Lu, E. Paganis, A. Psallidas, A. Steen, J. f. Tsai, B. Asavapibhop, K. Kovitanggoon, G. Singh, N. Srimanobhas, A. Bat, F. Boran, S. Damarseckin, Z. S. Demiroglu, C. Dozen, E. Eskut, S. Girgis, G. Gokbulut, Y. Guler, I. Hos, E. E. Kangal, O. Kara, A. Kayis Topaksu, U. Kiminsu, M. Oglakci, G. Onengut, K. Ozdemir, S. Ozturk, A. Polatoz, B. Tali, U. G. Tok, S. Turkcapar, I. S. Zorbakir, C. Zorbilmez, G. Karapinar, K. Ocalan, M. Yalvac, M. Zeyrek, E. Gülmez, M. Kaya, O. Kaya, S. Tekten, E. A. Yetkin, M. N. Agaras, S. Atay, A. Cakir, K. Cankocak, Y. Komurcu, B. Grynyov, L. Levchuk, F. Ball, L. Beck, J. J. Brooke, D. Burns, E. Clement, D. Cussans, O. Davignon, H. Flacher, J. Goldstein, G. P. Heath, H. F. Heath, L. Kreczko, D. M. Newbold, S. Paramesvaran, T. Sakuma, S. Seif El Nasr-storey, D. Smith, V. J. Smith, K. W. Bell, A. Belyaev, C. Brew, R. M. Brown, L. Calligaris, D. Cieri, D. J. A. Cockerill, J. A. Coughlan, K. Harder, S. Harper, J. Linacre, E. Olaiya, D. Petyt, C. H. Shepherd-Themistocleous, A. Thea, I. R. Tomalin, T. Williams, W. J. Womersley, G. Auzinger, R. Bainbridge, P. Bloch, J. Borg, S. Breeze, O. Buchmuller, A. Bundock, S. Casasso, D. Colling, L. Corpe, P. Dauncey, G. Davies, M. Della Negra, R. Di Maria, Y. Haddad, G. Hall, G. Iles, T. James, M. Komm, R. Lane, C. Laner, L. Lyons, A.-M. Magnan, S. Malik, L. Mastrolorenzo, T. Matsushita, J. Nash, A. Nikitenko, V. Palladino, M. Pesaresi, A. Richards, A. Rose, E. Scott, C. Seez, A. Shtipliyski, T. Strebler, S. Summers, A. Tapper, K. Uchida, M. Vazquez Acosta, T. Virdee, N. Wardle, D. Winterbottom, J. Wright, S. C. Zenz, J. E. Cole, P. R. Hobson, A. Khan, P. Kyberd, A. Morton, I. D. Reid, L. Teodorescu, S. Zahid, A. Borzou, K. Call, J. Dittmann, K. Hatakeyama, H. Liu, N. Pastika, C. Smith, R. Bartek, A. Dominguez, A. Buccilli, S. I. Cooper, C. Henderson, P. Rumerio, C. West, D. Arcaro, A. Avetisyan, T. Bose, D. Gastler, D. Rankin, C. Richardson, J. Rohlf, L. Sulak, D. Zou, G. Benelli, D. Cutts, M. Hadley, J. Hakala, U. Heintz, J. M. Hogan, K. H. M. Kwok, E. Laird, G. Landsberg, J. Lee, Z. Mao, M. Narain, J. Pazzini, S. Piperov, S. Sagir, R. Syarif, D. Yu, R. Band, C. Brainerd, R. Breedon, D. Burns, M. Calderon De La Barca Sanchez, M. Chertok, J. Conway, R. Conway, P. T. Cox, R. Erbacher, C. Flores, G. Funk, W. Ko, R. Lander, C. Mclean, M. Mulhearn, D. Pellett, J. Pilot, S. Shalhout, M. Shi, J. Smith, D. Stolp, D. Taylor, K. Tos, M. Tripathi, Z. Wang, F. Zhang, M. Bachtis, C. Bravo, R. Cousins, A. Dasgupta, A. Florent, J. Hauser, M. Ignatenko, N. Mccoll, S. Regnard, D. Saltzberg, C. Schnaible, V. Valuev, E. Bouvier, K. Burt, R. Clare, J. Ellison, J. W. Gary, S. M. A. Ghiasi Shirazi, G. Hanson, G. Karapostoli, E. Kennedy, F. Lacroix, O. R. Long, M. Olmedo Negrete, M. I. Paneva, W. Si, L. Wang, H. Wei, S. Wimpenny, B. R. Yates, J. G. Branson, S. Cittolin, M. Derdzinski, R. Gerosa, D. Gilbert, B. Hashemi, A. Holzner, D. Klein, G. Kole, V. Krutelyov, J. Letts, M. Masciovecchio, D. Olivito, S. Padhi, M. Pieri, M. Sani, V. Sharma, S. Simon, M. Tadel, A. Vartak, S. Wasserbaech, J. Wood, F. Würthwein, A. Yagil, G. Zevi Della Porta, N. Amin, R. Bhandari, J. Bradmiller-Feld, C. Campagnari, M. Citron, A. Dishaw, V. Dutta, M. Franco Sevilla, L. Gouskos, R. Heller, J. Incandela, A. Ovcharova, H. Qu, J. Richman, D. Stuart, I. Suarez, J. Yoo, D. Anderson, A. Bornheim, J. Bunn, J. M. Lawhorn, H. B. Newman, T. Q. Nguyen, C. Pena, M. Spiropulu, J. R. Vlimant, R. Wilkinson, S. Xie, Z. Zhang, R. Y. Zhu, M. B. Andrews, T. Ferguson, T. Mudholkar, M. Paulini, J. Russ, M. Sun, H. Vogel, I. Vorobiev, M. Weinberg, J. P. Cumalat, W. T. Ford, F. Jensen, A. Johnson, M. Krohn, S. Leontsinis, E. Macdonald, T. Mulholland, K. Stenson, K. A. Ulmer, S. R. Wagner, J. Alexander, J. Chaves, Y. Cheng, J. Chu, A. Datta, S. Dittmer, K. Mcdermott, N. Mirman, J. R. Patterson, D. Quach, A. Rinkevicius, A. Ryd, L. Skinnari, L. Soffi, S. M. Tan, Z. Tao, J. Thom, J. Tucker, P. Wittich, M. Zientek, S. Abdullin, M. Albrow, M. Alyari, G. Apollinari, A. Apresyan, A. Apyan, S. Banerjee, L. A. T. Bauerdick, A. Beretvas, J. Berryhill, P. C. Bhat, G. Bolla, K. Burkett, J. N. Butler, A. Canepa, G. B. Cerati, H. W. K. Cheung, F. Chlebana, M. Cremonesi, J. Duarte, V. D. Elvira, J. Freeman, Z. Gecse, E. Gottschalk, L. Gray, D. Green, S. Grünendahl, O. Gutsche, J. Hanlon, R. M. Harris, S. Hasegawa, J. Hirschauer, Z. Hu, B. Jayatilaka, S. Jindariani, M. Johnson, U. Joshi, B. Klima, B. Kreis, S. Lammel, D. Lincoln, R. Lipton, M. Liu, T. Liu, R. Lopes De Sá, J. Lykken, K. Maeshima, N. Magini, J. M. Marraffino, D. Mason, P. McBride, P. Merkel, S. Mrenna, S. Nahn, V. O’Dell, K. Pedro, O. Prokofyev, G. Rakness, L. Ristori, A. Savoy-Navarro, B. Schneider, E. Sexton-Kennedy, A. Soha, W. J. Spalding, L. Spiegel, S. Stoynev, J. Strait, N. Strobbe, L. Taylor, S. Tkaczyk, N. V. Tran, L. Uplegger, E. W. Vaandering, C. Vernieri, M. Verzocchi, R. Vidal, M. Wang, H. A. Weber, A. Whitbeck, W. Wu, D. Acosta, P. Avery, P. Bortignon, D. Bourilkov, A. Brinkerhoff, A. Carnes, M. Carver, D. Curry, R. D. Field, I. K. Furic, S. V. Gleyzer, B. M. Joshi, J. Konigsberg, A. Korytov, K. Kotov, P. Ma, K. Matchev, H. Mei, G. Mitselmakher, K. Shi, D. Sperka, N. Terentyev, L. Thomas, J. Wang, S. Wang, J. Yelton, Y. R. Joshi, S. Linn, P. Markowitz, J. L. Rodriguez, A. Ackert, T. Adams, A. Askew, S. Hagopian, V. Hagopian, K. F. Johnson, T. Kolberg, G. Martinez, T. Perry, H. Prosper, A. Saha, A. Santra, V. Sharma, R. Yohay, M. M. Baarmand, V. Bhopatkar, S. Colafranceschi, M. Hohlmann, D. Noonan, T. Roy, F. Yumiceva, M. R. Adams, L. Apanasevich, D. Berry, R. R. Betts, R. Cavanaugh, X. Chen, O. Evdokimov, C. E. Gerber, D. A. Hangal, D. J. Hofman, K. Jung, J. Kamin, I. D. Sandoval Gonzalez, M. B. Tonjes, N. Varelas, H. Wang, Z. Wu, J. Zhang, B. Bilki, W. Clarida, K. Dilsiz, S. Durgut, R. P. Gandrajula, M. Haytmyradov, V. Khristenko, J.-P. Merlo, H. Mermerkaya, A. Mestvirishvili, A. Moeller, J. Nachtman, H. Ogul, Y. Onel, F. Ozok, A. Penzo, C. Snyder, E. Tiras, J. Wetzel, K. Yi, B. Blumenfeld, A. Cocoros, N. Eminizer, D. Fehling, L. Feng, A. V. Gritsan, P. Maksimovic, J. Roskes, U. Sarica, M. Swartz, M. Xiao, C. You, A. Al-bataineh, P. Baringer, A. Bean, S. Boren, J. Bowen, J. Castle, S. Khalil, A. Kropivnitskaya, D. Majumder, W. Mcbrayer, M. Murray, C. Rogan, C. Royon, S. Sanders, E. Schmitz, J. D. Tapia Takaki, Q. Wang, A. Ivanov, K. Kaadze, Y. Maravin, A. Mohammadi, L. K. Saini, N. Skhirtladze, F. Rebassoo, D. Wright, A. Baden, O. Baron, A. Belloni, S. C. Eno, Y. Feng, C. Ferraioli, N. J. Hadley, S. Jabeen, G. Y. Jeng, R. G. Kellogg, J. Kunkle, A. C. Mignerey, F. Ricci-Tam, Y. H. Shin, A. Skuja, S. C. Tonwar, D. Abercrombie, B. Allen, V. Azzolini, R. Barbieri, A. Baty, G. Bauer, R. Bi, S. Brandt, W. Busza, I. A. Cali, M. D’Alfonso, Z. Demiragli, G. Gomez Ceballos, M. Goncharov, P. Harris, D. Hsu, M. Hu, Y. Iiyama, G. M. Innocenti, M. Klute, D. Kovalskyi, Y.-J. Lee, A. Levin, P. D. Luckey, B. Maier, A. C. Marini, C. Mcginn, C. Mironov, S. Narayanan, X. Niu, C. Paus, C. Roland, G. Roland, J. Salfeld-Nebgen, G. S. F. Stephans, K. Sumorok, K. Tatar, D. Velicanu, J. Wang, T. W. Wang, B. Wyslouch, S. Zhaozhong, A. C. Benvenuti, R. M. Chatterjee, A. Evans, P. Hansen, S. Kalafut, Y. Kubota, Z. Lesko, J. Mans, S. Nourbakhsh, N. Ruckstuhl, R. Rusack, J. Turkewitz, M. A. Wadud, J. G. Acosta, S. Oliveros, E. Avdeeva, K. Bloom, D. R. Claes, C. Fangmeier, F. Golf, R. Gonzalez Suarez, R. Kamalieddin, I. Kravchenko, J. Monroy, J. E. Siado, G. R. Snow, B. Stieger, J. Dolen, A. Godshalk, C. Harrington, I. Iashvili, D. Nguyen, A. Parker, S. Rappoccio, B. Roozbahani, G. Alverson, E. Barberis, C. Freer, A. Hortiangtham, A. Massironi, D. M. Morse, T. Orimoto, R. Teixeira De Lima, T. Wamorkar, B. Wang, A. Wisecarver, D. Wood, S. Bhattacharya, O. Charaf, K. A. Hahn, N. Mucia, N. Odell, M. H. Schmitt, K. Sung, M. Trovato, M. Velasco, R. Bucci, N. Dev, M. Hildreth, K. Hurtado Anampa, C. Jessop, D. J. Karmgard, N. Kellams, K. Lannon, W. Li, N. Loukas, N. Marinelli, F. Meng, C. Mueller, Y. Musienko, M. Planer, A. Reinsvold, R. Ruchti, P. Siddireddy, G. Smith, S. Taroni, M. Wayne, A. Wightman, M. Wolf, A. Woodard, J. Alimena, L. Antonelli, B. Bylsma, L. S. Durkin, S. Flowers, B. Francis, A. Hart, C. Hill, W. Ji, T. Y. Ling, W. Luo, B. L. Winer, H. W. Wulsin, S. Cooperstein, O. Driga, P. Elmer, J. Hardenbrook, P. Hebda, S. Higginbotham, A. Kalogeropoulos, D. Lange, J. Luo, D. Marlow, K. Mei, I. Ojalvo, J. Olsen, C. Palmer, P. Piroué, D. Stickland, C. Tully, S. Malik, S. Norberg, A. Barker, V. E. Barnes, S. Das, L. Gutay, M. Jones, A. W. Jung, A. Khatiwada, D. H. Miller, N. Neumeister, C. C. Peng, H. Qiu, J. F. Schulte, J. Sun, F. Wang, R. Xiao, W. Xie, T. Cheng, N. Parashar, Z. Chen, K. M. Ecklund, S. Freed, F. J. M. Geurts, M. Guilbaud, M. Kilpatrick, W. Li, B. Michlin, B. P. Padley, J. Roberts, J. Rorie, W. Shi, Z. Tu, J. Zabel, A. Zhang, A. Bodek, P. de Barbaro, R. Demina, Y. t. Duh, T. Ferbel, M. Galanti, A. Garcia-Bellido, J. Han, O. Hindrichs, A. Khukhunaishvili, K. H. Lo, P. Tan, M. Verzetti, R. Ciesielski, K. Goulianos, C. Mesropian, A. Agapitos, J. P. Chou, Y. Gershtein, T. A. Gómez Espinosa, E. Halkiadakis, M. Heindl, E. Hughes, S. Kaplan, R. Kunnawalkam Elayavalli, S. Kyriacou, A. Lath, R. Montalvo, K. Nash, M. Osherson, H. Saka, S. Salur, S. Schnetzer, D. Sheffield, S. Somalwar, R. Stone, S. Thomas, P. Thomassen, M. Walker, A. G. Delannoy, J. Heideman, G. Riley, K. Rose, S. Spanier, K. Thapa, O. Bouhali, A. Castaneda Hernandez, A. Celik, M. Dalchenko, M. De Mattia, A. Delgado, S. Dildick, R. Eusebi, J. Gilmore, T. Huang, T. Kamon, R. Mueller, Y. Pakhotin, R. Patel, A. Perloff, L. Perniè, D. Rathjens, A. Safonov, A. Tatarinov, N. Akchurin, J. Damgov, F. De Guio, P. R. Dudero, J. Faulkner, E. Gurpinar, S. Kunori, K. Lamichhane, S. W. Lee, T. Mengke, S. Muthumuni, T. Peltola, S. Undleeb, I. Volobouev, Z. Wang, S. Greene, A. Gurrola, R. Janjam, W. Johns, C. Maguire, A. Melo, H. Ni, K. Padeken, P. Sheldon, S. Tuo, J. Velkovska, Q. Xu, M. W. Arenton, P. Barria, B. Cox, R. Hirosky, M. Joyce, A. Ledovskoy, H. Li, C. Neu, T. Sinthuprasith, Y. Wang, E. Wolfe, F. Xia, R. Harr, P. E. Karchin, N. Poudyal, J. Sturdy, P. Thapa, S. Zaleski, M. Brodski, J. Buchanan, C. Caillol, D. Carlsmith, S. Dasu, L. Dodd, S. Duric, B. Gomber, M. Grothe, M. Herndon, A. Hervé, U. Hussain, P. Klabbers, A. Lanaro, A. Levine, K. Long, R. Loveless, V. Rekovic, T. Ruggles, A. Savin, N. Smith, W. H. Smith, N. Woods, CMS Collaboration

**Affiliations:** 1 Yerevan Physics Institute, Yerevan, Armenia; 2 Institut für Hochenergiephysik, Vienna, Austria; 3 Institute for Nuclear Problems, Minsk, Belarus; 4 Universiteit Antwerpen, Antwerpen, Belgium; 5 Vrije Universiteit Brussel, Brussel, Belgium; 6 Université Libre de Bruxelles, Bruxelles, Belgium; 7 Ghent University, Ghent, Belgium; 8 Université Catholique de Louvain, Louvain-la-Neuve, Belgium; 9 Centro Brasileiro de Pesquisas Fisicas, Rio de Janeiro, Brazil; 10 Universidade do Estado do Rio de Janeiro, Rio de Janeiro, Brazil; 11 Universidade Estadual Paulista , Universidade Federal do ABC, São Paulo, Brazil; 12 Institute for Nuclear Research and Nuclear Energy, Bulgarian Academy of Sciences, Sofia, Bulgaria; 13 University of Sofia, Sofia, Bulgaria; 14 Beihang University, Beijing, China; 15 Institute of High Energy Physics, Beijing, China; 16 State Key Laboratory of Nuclear Physics and Technology, Peking University, Beijing, China; 17 Tsinghua University, Beijing, China; 18 Universidad de Los Andes, Bogota, Colombia; 19 University of Split, Faculty of Electrical Engineering, Mechanical Engineering and Naval Architecture, Split, Croatia; 20 University of Split, Faculty of Science, Split, Croatia; 21 Institute Rudjer Boskovic, Zagreb, Croatia; 22 University of Cyprus, Nicosia, Cyprus; 23 Charles University, Prague, Czech Republic; 24 Universidad San Francisco de Quito, Quito, Ecuador; 25 Academy of Scientific Research and Technology of the Arab Republic of Egypt, Egyptian Network of High Energy Physics, Cairo, Egypt; 26 National Institute of Chemical Physics and Biophysics, Tallinn, Estonia; 27 Department of Physics, University of Helsinki, Helsinki, Finland; 28 Helsinki Institute of Physics, Helsinki, Finland; 29 Lappeenranta University of Technology, Lappeenranta, Finland; 30 IRFU, CEA, Université Paris-Saclay, Gif-sur-Yvette, France; 31 Laboratoire Leprince-Ringuet, Ecole polytechnique, CNRS/IN2P3, Université Paris-Saclay, Palaiseau, France; 32 Université de Strasbourg, CNRS, IPHC UMR 7178, 67000 Strasbourg, France; 33 Centre de Calcul de l’Institut National de Physique Nucleaire et de Physique des Particules, CNRS/IN2P3, Villeurbanne, France; 34 Université de Lyon, Université Claude Bernard Lyon 1, CNRS-IN2P3, Institut de Physique Nucléaire de Lyon, Villeurbanne, France; 35 Georgian Technical University, Tbilisi, Georgia; 36 Tbilisi State University, Tbilisi, Georgia; 37 RWTH Aachen University, I. Physikalisches Institut, Aachen, Germany; 38 RWTH Aachen University, III. Physikalisches Institut A, Aachen, Germany; 39 RWTH Aachen University, III. Physikalisches Institut B, Aachen, Germany; 40 Deutsches Elektronen-Synchrotron, Hamburg, Germany; 41 University of Hamburg, Hamburg, Germany; 42 Institut für Experimentelle Teilchenphysik, Karlsruhe, Germany; 43 Institute of Nuclear and Particle Physics (INPP), NCSR Demokritos, Aghia Paraskevi, Greece; 44 National and Kapodistrian University of Athens, Athens, Greece; 45 National Technical University of Athens, Athens, Greece; 46 University of Ioánnina, Ioánnina, Greece; 47 MTA-ELTE Lendület CMS Particle and Nuclear Physics Group, Eötvös Loránd University, Budapest, Hungary; 48 Wigner Research Centre for Physics, Budapest, Hungary; 49 Institute of Nuclear Research ATOMKI, Debrecen, Hungary; 50 Institute of Physics, University of Debrecen, Debrecen, Hungary; 51 Indian Institute of Science (IISc), Bangalore, India; 52 National Institute of Science Education and Research, Bhubaneswar, India; 53 Panjab University, Chandigarh, India; 54 University of Delhi, Delhi, India; 55 Saha Institute of Nuclear Physics, HBNI, Kolkata, India; 56 Indian Institute of Technology Madras, Madras, India; 57 Bhabha Atomic Research Centre, Mumbai, India; 58 Tata Institute of Fundamental Research-A, Mumbai, India; 59 Tata Institute of Fundamental Research-B, Mumbai, India; 60 Indian Institute of Science Education and Research (IISER), Pune, India; 61 Institute for Research in Fundamental Sciences (IPM), Tehran, Iran; 62 University College Dublin, Dublin, Ireland; 63 INFN Sezione di Bari , Università di Bari , Politecnico di Bari, Bari, Italy; 64 INFN Sezione di Bologna , Università di Bologna, Bologna, Italy; 65 INFN Sezione di Catania , Università di Catania, Catania, Italy; 66 INFN Sezione di Firenze , Università di Firenze, Firenze, Italy; 67 INFN Laboratori Nazionali di Frascati, Frascati, Italy; 68 INFN Sezione di Genova , Università di Genova, Genoa, Italy; 69 INFN Sezione di Milano-Bicocca , Università di Milano-Bicocca, Milan, Italy; 70 INFN Sezione di Napoli , Università di Napoli ’Federico II’ , Napoli, Italy, Università della Basilicata , Potenza, Italy, Università G. Marconi, Rome, Italy; 71 INFN Sezione di Padova , Università di Padova , Padova, Italy, Università di Trento, Trento, Italy; 72 INFN Sezione di Pavia , Università di Pavia, Pavia, Italy; 73 INFN Sezione di Perugia , Università di Perugia, Perugia, Italy; 74 INFN Sezione di Pisa , Università di Pisa , Scuola Normale Superiore di Pisa, Pisa, Italy; 75 INFN Sezione di Roma , Sapienza Università di Roma, Rome, Italy; 76 INFN Sezione di Torino , Università di Torino , Torino, Italy, Università del Piemonte Orientale, Novara, Italy; 77 INFN Sezione di Trieste , Università di Trieste, Trieste, Italy; 78 Kyungpook National University, Daegu, South Korea; 79 Chonnam National University, Institute for Universe and Elementary Particles, Kwangju, Korea; 80 Hanyang University, Seoul, Korea; 81 Korea University, Seoul, Korea; 82 Seoul National University, Seoul, Korea; 83 University of Seoul, Seoul, Korea; 84 Sungkyunkwan University, Suwon, Korea; 85 Vilnius University, Vilnius, Lithuania; 86 National Centre for Particle Physics, Universiti Malaya, Kuala Lumpur, Malaysia; 87 Centro de Investigacion y de Estudios Avanzados del IPN, Mexico City, Mexico; 88 Universidad Iberoamericana, Mexico City, Mexico; 89 Benemerita Universidad Autonoma de Puebla, Puebla, Mexico; 90 Universidad Autónoma de San Luis Potosí, San Luis Potosí, Mexico; 91 University of Auckland, Auckland, New Zealand; 92 University of Canterbury, Christchurch, New Zealand; 93 National Centre for Physics, Quaid-I-Azam University, Islamabad, Pakistan; 94 National Centre for Nuclear Research, Swierk, Poland; 95 Institute of Experimental Physics, Faculty of Physics, University of Warsaw, Warsaw, Poland; 96 Laboratório de Instrumentação e Física Experimental de Partículas, Lisbon, Portugal; 97 Joint Institute for Nuclear Research, Dubna, Russia; 98 Petersburg Nuclear Physics Institute, Gatchina (St. Petersburg), Russia; 99 Institute for Nuclear Research, Moscow, Russia; 100 Institute for Theoretical and Experimental Physics, Moscow, Russia; 101 Moscow Institute of Physics and Technology, Moscow, Russia; 102 National Research Nuclear University ’Moscow Engineering Physics Institute’ (MEPhI), Moscow, Russia; 103 P.N. Lebedev Physical Institute, Moscow, Russia; 104 Skobeltsyn Institute of Nuclear Physics, Lomonosov Moscow State University, Moscow, Russia; 105 Novosibirsk State University (NSU), Novosibirsk, Russia; 106 State Research Center of Russian Federation, Institute for High Energy Physics of NRC&quot, Kurchatov Institute&quot, Protvino, Russia; 107 National Research Tomsk Polytechnic University, Tomsk, Russia; 108 University of Belgrade, Faculty of Physics and Vinca Institute of Nuclear Sciences, Belgrade, Serbia; 109 Centro de Investigaciones Energéticas Medioambientales y Tecnológicas (CIEMAT), Madrid, Spain; 110 Universidad Autónoma de Madrid, Madrid, Spain; 111 Universidad de Oviedo, Oviedo, Spain; 112 Instituto de Física de Cantabria (IFCA), CSIC-Universidad de Cantabria, Santander, Spain; 113 CERN, European Organization for Nuclear Research, Geneva, Switzerland; 114 Paul Scherrer Institut, Villigen, Switzerland; 115 ETH Zurich, Institute for Particle Physics and Astrophysics (IPA), Zurich, Switzerland; 116 Universität Zürich, Zurich, Switzerland; 117 National Central University, Chung-Li, Taiwan; 118 National Taiwan University (NTU), Taipei, Taiwan; 119 Chulalongkorn University, Faculty of Science, Department of Physics, Bangkok, Thailand; 120 Çukurova University, Physics Department, Science and Art Faculty, Adana, Turkey; 121 Middle East Technical University, Physics Department, Ankara, Turkey; 122 Bogazici University, Istanbul, Turkey; 123 Istanbul Technical University, Istanbul, Turkey; 124 Institute for Scintillation Materials of National Academy of Science of Ukraine, Kharkov, Ukraine; 125 National Scientific Center, Kharkov Institute of Physics and Technology, Kharkov, Ukraine; 126 University of Bristol, Bristol, UK; 127 Rutherford Appleton Laboratory, Didcot, UK; 128 Imperial College, London, UK; 129 Brunel University, Uxbridge, UK; 130 Baylor University, Waco, USA; 131 Catholic University of America, Washington DC, USA; 132 The University of Alabama, Tuscaloosa, USA; 133 Boston University, Boston, USA; 134 Brown University, Providence, USA; 135 University of California, Davis, Davis USA; 136 University of California, Los Angeles, USA; 137 University of California, Riverside, Riverside USA; 138 University of California, San Diego, La Jolla USA; 139 University of California, Santa Barbara, Department of Physics, Santa Barbara, USA; 140 California Institute of Technology, Pasadena, USA; 141 Carnegie Mellon University, Pittsburgh, USA; 142 University of Colorado Boulder, Boulder, USA; 143 Cornell University, Ithaca, USA; 144 Fermi National Accelerator Laboratory, Batavia, USA; 145 University of Florida, Gainesville, USA; 146 Florida International University, Miami, USA; 147 Florida State University, Tallahassee, USA; 148 Florida Institute of Technology, Melbourne, USA; 149 University of Illinois at Chicago (UIC), Chicago, USA; 150 The University of Iowa, Iowa City, USA; 151 Johns Hopkins University, Baltimore, USA; 152 The University of Kansas, Lawrence, USA; 153 Kansas State University, Manhattan, USA; 154 Lawrence Livermore National Laboratory, Livermore, USA; 155 University of Maryland, College Park, USA; 156 Massachusetts Institute of Technology, Cambridge, USA; 157 University of Minnesota, Minneapolis, USA; 158 University of Mississippi, Oxford, USA; 159 University of Nebraska-Lincoln, Lincoln, USA; 160 State University of New York at Buffalo, Buffalo, USA; 161 Northeastern University, Boston, USA; 162 Northwestern University, Evanston, USA; 163 University of Notre Dame, Notre Dame, USA; 164 The Ohio State University, Columbus, USA; 165 Princeton University, Princeton, USA; 166 University of Puerto Rico, Mayaguez, USA; 167 Purdue University, West Lafayette, USA; 168 Purdue University Northwest, Hammond, USA; 169 Rice University, Houston, USA; 170 University of Rochester, Rochester, USA; 171 The Rockefeller University, New York, USA; 172 Rutgers, The State University of New Jersey, Piscataway, USA; 173 University of Tennessee, Knoxville, USA; 174 Texas A&M University, College Station, USA; 175 Texas Tech University, Lubbock, USA; 176 Vanderbilt University, Nashville, USA; 177 University of Virginia, Charlottesville, USA; 178 Wayne State University, Detroit, USA; 179 University of Wisconsin, Madison, Madison, WI USA; 180 CERN, 1211 Geneva 23, Switzerland

## Abstract

A search for pair production of heavy scalar leptoquarks (LQs), each decaying into a top quark and a $\tau$ lepton, is presented. The search considers final states with an electron or a muon, one or two $\tau$ leptons that decayed to hadrons, and additional jets. The data were collected in 2016 in proton–proton collisions at $\sqrt{s}=13\,\text {Te}\text {V}$ with the CMS detector at the LHC, and correspond to an integrated luminosity of 35.9$\,\text {fb}^{-1}$. No evidence for pair production of LQs is found. Assuming a branching fraction of unity for the decay $\mathrm {LQ} \rightarrow \mathrm {t}\tau$, upper limits on the production cross section are set as a function of LQ mass, excluding masses below 900$\,\text {Ge}\text {V}$ at 95% confidence level. These results provide the most stringent limits to date on the production of scalar LQs that decay to a top quark and a $\tau$ lepton.

## Introduction

Leptoquarks (LQs) are hypothetical particles that carry non-zero baryon and lepton quantum numbers. They are charged under all standard model (SM) gauge groups, and their possible quantum numbers can be restricted by the assumption that their interactions with SM fermions are renormalizable and gauge invariant [1]. The spin of an LQ state is either 0 (scalar LQ) or 1 (vector LQ). Leptoquarks appear in theories beyond the SM such as grand unified theories [2–4], technicolor models [5, 6] and other compositeness scenarios [7, 8], and R-parity-violating (RPV) supersymmetric models [9, 10].

Third-generation scalar LQs ($\mathrm {LQ}_3$ s) have recently received considerable theoretical interest, as their existence can explain the anomaly in the $\mathrm {\overline{B}}\rightarrow \mathrm {D}\tau \overline{\nu }$ and $\mathrm {\overline{B}}\rightarrow \mathrm {D}^* \tau \overline{\nu }$ decay rates reported by the BaBar [11, 12], Belle [13–15], and LHCb [16] Collaborations. These decay rates deviate from the SM predictions by about four standard deviations [17], and studies of the flavor structure of LQ couplings reveal that large couplings to third-generation quarks and leptons could explain this anomaly [18–21]. Third-generation LQs can appear in models in which only third-generation quarks and leptons are unified [22, 23] and therefore their existence is not constrained by proton decay experiments. All models that predict LQs with masses at the TeV scale and sizable couplings to top quarks and $\tau$ leptons can be probed by the CMS experiment at the CERN LHC.

**Fig. 1:**
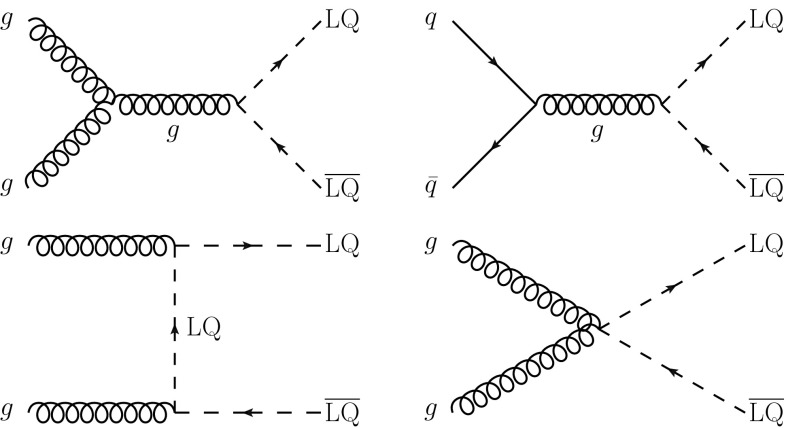
Dominant leading order Feynman diagrams for the production of leptoquark pairs in proton–proton collisions

In proton–proton ($\mathrm {p}\mathrm {p}$) collisions LQs are mainly pair produced through the quantum chromodynamic (QCD) quark-antiquark annihilation and gluon-gluon fusion *s*- and *t*-channel subprocesses as shown in Fig. 1. There are also lepton-mediated *t*- and *u*-channel contributions that depend on the unknown lepton-quark-LQ Yukawa coupling, but these contributions to $\mathrm {LQ}_3$ production are negligible at the LHC as they require third-generation quarks in the initial state. Hence, the LQ pair-production cross section can be taken to depend only on the assumed values of the LQ spin and mass, and on the center-of-mass energy. The corresponding pair production cross sections have been calculated up to next-to-leading order (NLO) in perturbative QCD [24].

This paper presents the first search for the production of an $\mathrm {LQ}_3$ decaying into a top quark and a $\tau$ lepton at $\sqrt{s} = 13\,\text {Te}\text {V}$. The search targets $\mathrm {LQ}_3$ s with electric charges $-5/3\,e$ and $-1/3\,e$, where *e* is the proton charge, and with various possible weak isospin configurations, depending on the model. A previous search for this channel at $\sqrt{s} = 8\,\text {Te}\text {V}$ by the CMS Collaboration resulted in a lower mass limit of 685$\,\text {Ge}\text {V}$ for an $\mathrm {LQ}_3$ with branching fraction $\mathcal {B}=1$ into a top quark and a $\tau$ lepton [25]. Other searches for an $\mathrm {LQ}_3$ have targeted the decays $\mathrm {LQ}_3 \rightarrow \mathrm {b}\nu$ and $\mathrm {LQ}_3 \rightarrow \mathrm {b}\tau$ [26–39]. The results of the search presented here are also interpreted in the context of RPV supersymmetric models, where the supersymmetric partner of the bottom quark (bottom squark) decays into a top quark and a $\tau$ lepton via the RPV coupling.

We consider events with at least one electron or muon and at least one $\tau$ lepton, where the $\tau$ lepton undergoes a one- or three-prong hadronic decay, $\tau _\mathrm {h} \rightarrow \text {hadron(s)}+\nu _\tau$. In $\mathrm {LQ}_3 \overline{\mathrm {LQ}}_3$ events, $\tau$ leptons arise directly from $\mathrm {LQ}_3$ decays, as well as from $\mathrm {W}$ bosons in the top quark decay chain. Electrons and muons are produced in leptonic decays of $\mathrm {W}$ bosons or $\tau$ leptons. Two search regions are used in this analysis: a di-$\tau$ region with the signature $\ell \tau _\mathrm {h} \tau _\mathrm {h}$+jets and small background levels from SM processes, which provides high sensitivity for $\mathrm {LQ}_3$ masses below 500$\,\text {Ge}\text {V}$, and a region with a single $\tau$ lepton in the final state, $\ell \tau _\mathrm {h}$+jets, which has higher sensitivity for $\mathrm {LQ}_3$ masses above 500$\,\text {Ge}\text {V}$ because of a larger signal efficiency. Here, $\ell$ denotes either an electron or a muon. The dominant backgrounds in this search come from ${\mathrm {t}\overline{\mathrm {t}}}$+jets and $\mathrm {W}+\text {jets}$ production, with jets misidentified as hadronically decaying $\tau$ leptons. These backgrounds are estimated through measurements in control regions and extrapolated to the signal region.

In this paper, Sect. 2 describes the CMS detector, while Sect. 3 discusses the data samples and the properties of simulated events utilized in the analysis. Section 4 outlines the techniques used for event reconstruction and Sect. 5 describes the selection criteria applied in each analysis channel. The method used for the background estimation is reported in Sect. 6, and systematic uncertainties are detailed in Sect. 7. Finally, Sect. 8 contains the results of the analysis, and Sect. 9 summarizes this work.

## The CMS detector

The central feature of the CMS apparatus [40] is a superconducting solenoid of 6$\,\text {m}$ internal diameter, providing a magnetic field of 3.8$\,\text {T}$. Within the solenoid volume are a silicon pixel and strip tracker, a lead tungstate crystal electromagnetic calorimeter (ECAL), and a brass and scintillator hadron calorimeter (HCAL), each composed of a barrel and two endcap sections. Forward calorimeters extend the pseudorapidity ($\eta$) coverage provided by the barrel and endcap detectors. Electron momenta are estimated by combining the energy measurement in the ECAL with the momentum measurement in the tracker. Muons are measured in gas-ionization detectors embedded in the steel flux-return yoke outside the solenoid. A more detailed description of the CMS detector, together with a definition of the coordinate system used and the relevant kinematic variables, can be found in Ref. [40].

Events of interest are selected using a two-tiered trigger system [41], where the first level is composed of custom hardware processors and selects events at a rate of around 100$\,\text {kHz}$ within a time interval of less than 4$\,\upmu \text {s}$. The second level, known as the high-level trigger, uses a version of the full event reconstruction software optimized for fast processing, and reduces the event rate to around 1$\,\text {kHz}$ before data storage.

## Data sample and simulated events

The search for $\mathrm {LQ}_3$ s presented here uses $\mathrm {p}\mathrm {p}$ collisions at $\sqrt{s}=13\,\text {Te}\text {V}$ recorded with the CMS detector in 2016. The data sample corresponds to an integrated luminosity of 35.9$\,\text {fb}^{-1}$  [42].

The leading order (LO) Monte Carlo (MC) program pythia  8.205 [43] is used to simulate the $\mathrm {LQ}_3$ pair production signal process. Both $\mathrm {LQ}_3$ s are required to decay into a top quark and a $\tau$ lepton, and polarization effects from the chiralities of the top quark and the $\tau$ lepton have been neglected. The signal samples are generated for $\mathrm {LQ}_3$ masses ranging from 200 to 2000$\,\text {Ge}\text {V}$.

The principal background processes, top quark pair production (${\mathrm {t}\overline{\mathrm {t}}}$) via the strong interaction and electroweak single top quark production in the *t*-channel and tW processes, are simulated with the NLO generator powheg (v1 is used for the single top $\mathrm {t}$ $\mathrm {W}$ processes and v2 for the single top *t*-channel and ${\mathrm {t}\overline{\mathrm {t}}}$ processes) [44–49]. The *s*-channel process of single top quark production is generated at NLO using the program MadGraph 5_amc@nlo  (v2.2.2) [50]. Other background processes involve $\mathrm {W}$ and $\mathrm {Z}$ boson production in association with jet radiation. These processes are generated with MadGraph 5_amc@nlo  (v2.2.2), with $\mathrm {W}$ boson production at NLO and $\mathrm {Z}$ boson production at LO level. The matrix element generation of $\mathrm {W}$ and $\mathrm {Z}$ boson production is matched to the parton shower emissions with the Frederix and Frixione [51] and MLM [52] algorithms, respectively. Background processes from QCD multijet production are simulated with pythia  8.205. For all generated events, pythia  8.205 is used for the description of the parton shower and hadronization. In the parton shower, the underlying event tune CUETP8M1 [53, 54] has been applied for all samples except for ${\mathrm {t}\overline{\mathrm {t}}}$ and single top quark production in the *t*-channel, which use the underlying event tune CUETP8M2T4 [53, 54]. The event generation is performed using the NNPDF 3.0 parton distribution functions (PDFs) [55], for all events. The detector response is modeled with the Geant4  [56] suite of programs.

## Event reconstruction

Event reconstruction is based on the CMS particle-flow (PF) algorithm [57], which combines information from all subdetectors, including measurements from the tracking system, energy deposits in the ECAL and HCAL, and tracks reconstructed in the muon detectors. Based on this information, all particles in the event are reconstructed as electrons, muons, photons, charged hadrons, or neutral hadrons.

Interaction vertices are reconstructed using a deterministic annealing filtering algorithm [58, 59]. The reconstructed vertex with the largest value of summed physics-objects $p_{\mathrm {T}} ^2$ is taken to be the primary $\mathrm {p}\mathrm {p}$ interaction vertex. The physics objects are jets, clustered using the jet finding algorithm [60, 61] with the tracks assigned to the vertex as inputs, and the associated missing transverse momentum, taken as the negative vector sum of the $p_{\mathrm {T}}$ of those jets. Charged particles associated with other interaction vertices are removed from further consideration.

Muons are reconstructed using the information collected in the muon detectors and the inner tracking detectors, and are measured in the range $|\eta |< 2.4$. Tracks associated with muon candidates must be consistent with muons originating from the primary vertex, and are required to satisfy a set of identification requirements. Matching muon detector information to tracks measured in the silicon tracker results in a $p_{\mathrm {T}}$ resolution for muons with $20<p_{\mathrm {T}} < 100\,\text {Ge}\text {V}$ of 1.3–2.0% in the barrel and better than 6% in the endcaps. The $p_{\mathrm {T}}$ resolution in the barrel is better than 10% for muons with $p_{\mathrm {T}}$ up to 1$\,\text {Te}\text {V}$  [62].

Electron candidates are reconstructed in the range $|\eta |<2.5$ by combining tracking information with energy deposits in the ECAL. Candidates are identified [63] using information on the spatial distribution of the shower, the track quality and the spatial match between the track and electromagnetic cluster, the fraction of total cluster energy in the HCAL, and the level of activity in the surrounding tracker and calorimeter regions. The transverse momentum $p_{\mathrm {T}}$ resolution for electrons with $p_{\mathrm {T}} \approx 45\,\text {Ge}\text {V}$ from $\mathrm {Z} \rightarrow \mathrm {e}\mathrm {e}$ decays ranges from 1.7% for nonshowering electrons in the barrel region to 4.5% for electrons showering in the endcaps [63].

Jets are clustered using PF candidates as inputs to the anti-$k_{\mathrm {T}}$ algorithm [60] in the FastJet  3.0 software package [61], using a distance parameter of 0.4. For all jets, corrections based on the jet area [64] are applied to the energy of the jets to remove the energy contributions from neutral hadrons from additional pp interactions in the same or adjacent bunch crossings (pileup collisions). Subsequent corrections are used to account for the nonlinear calorimetric response in both jet energy and mass, as a function of $\eta$ and $p_{\mathrm {T}}$ [65]. The jet energy resolution amounts typically to 15% at 10$\,\text {Ge}\text {V}$, 8% at 100$\,\text {Ge}\text {V}$, and 4% at 1$\,\text {Te}\text {V}$  [66]. Corrections to the jet energy scale and the jet energy resolution are propagated to the determination of the missing transverse momentum [66]. Jets associated with $\mathrm {b}$ quarks are identified using the combined secondary vertex v2 algorithm [67, 68]. The working point used for jet $\mathrm {b}$ tagging in this analysis has an efficiency of $\approx$65% (in ${\mathrm {t}\overline{\mathrm {t}}}$ simulated events) and a mistag rate (the rate at which light-flavor jets are incorrectly tagged) of approximately 1% [68].

Hadronically decaying $\tau$ leptons are reconstructed with the hadron-plus-strips (HPS) algorithm [69] and are denoted by $\tau _\mathrm {h}$. The HPS algorithm is based on PF jets and additionally includes photons originating from neutral pion decays. Energy depositions in the ECAL are reconstructed in “strips” elongated in the direction of the azimuthal angle $\phi$, to take account of interactions in the material of the detector and the axial magnetic field. These deposits are associated with one or three charged tracks to reconstruct various hadronic decay modes of $\tau$ leptons. To suppress backgrounds from light-quark or gluon jets, a $\tau _\mathrm {h}$ candidate is required to be isolated from other energy deposits in the event. The isolation criterion is based on the scalar $p_{\mathrm {T}}$ sum $I_\tau$ of charged and neutral PF candidates within a cone of radius $\smash [b]{\sqrt{(\varDelta \eta )^2 + (\varDelta \phi )^2} = 0.5}$ around the $\tau _\mathrm {h}$ direction, excluding the $\tau _\mathrm {h}$ candidate. The isolation criterion is $I_\tau < 1.5\,\text {Ge}\text {V}$ [70].

The energies and resolutions as well as the selection efficiencies for all reconstructed jets and leptons are studied in data and simulated events [62, 63, 66, 68, 70]. Based on these studies, the simulation is corrected to match the data.

## Event selection and categorization

In the online trigger system, events with an isolated muon (or electron) with $p_{\mathrm {T}} >24\,(27)\,\text {Ge}\text {V}$ and $|\eta |<2.4\,(2.1)$ are selected in the muon (electron) channel. We select events offline containing exactly one isolated muon (or electron) with $p_{\mathrm {T}} >30\,\text {Ge}\text {V}$ and $|\eta |<2.4\,(2.1)$. For the electron channel, a veto is applied to events with a muon to avoid overlap between the two channels. At least one $\tau _\mathrm {h}$ lepton with $p_{\mathrm {T}} >20\,\text {Ge}\text {V}$ and $|\eta |<2.1$ and at least two jets with $p_{\mathrm {T}} >50\,\text {Ge}\text {V}$ and $|\eta | < 2.4$ are required. Events are selected if a third jet with $p_{\mathrm {T}} >30\,\text {Ge}\text {V}$ and $|\eta | < 2.4$ is present, and any additional jets are only considered if they have ${p_{\mathrm {T}} >30\,\text {Ge}\text {V}}$. The magnitude of the missing transverse momentum, $p_{\mathrm {T}} ^\text {miss}$, is required to be above 50$\,\text {Ge}\text {V}$. Further, the events are divided into two categories corresponding to the number of observed LQ candidates, allowing the sensitivity to be enhanced over a broad range of LQ masses. The event selection was chosen to maximize the expected significance of a possible LQ signal. A summary of the selection criteria for both categories is given in Table 1 and described below.

**Table 1:**
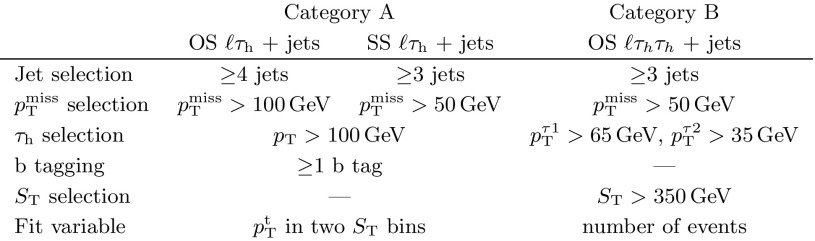
Summary of selection criteria in event categories A ($\ell \tau _\mathrm {h}$ + jets) and B ($\ell \tau _\mathrm {h} \tau _\mathrm {h}$ + jets), where $\ell = \mu , \mathrm {e}$. In category A, the two subcategories, OS and SS, are defined by the charge of the $\ell \tau _h$ pair. The fit variable used in each category is also shown

### Category A: $\ell \tau _\mathrm {h}$ + jets

In this category, exactly one $\tau _\mathrm {h}$ lepton is required in addition to the presence of one electron or muon. High $p_{\mathrm {T}}$ requirements are applied to maximize the sensitivity at high LQ masses. The leading jet is required to have ${p_{\mathrm {T}} >150\,\text {Ge}\text {V}}$. In addition we define two subcategories based on the electric charges of the particles in the $\ell \tau _\mathrm {h}$ pair: opposite-sign (OS) and same-sign (SS). Events passing the OS $\ell \tau _\mathrm {h}$ pair requirement must contain at least four jets and have $p_{\mathrm {T}} ^\text {miss} >100\,\text {Ge}\text {V}$. For both subcategories, we require that the leading tau lepton has $p_{\mathrm {T}} >100\,\text {Ge}\text {V}$ and that there is at least one $\mathrm {b}$-tagged jet. Finally the events are divided into two regions of $S_{\mathrm {T}}$, where $S_{\mathrm {T}}$ is the scalar $p_{\mathrm {T}}$ sum of all selected jets, leptons, and $p_{\mathrm {T}} ^\text {miss}$. In the low (high)-$S_{\mathrm {T}}$ search regions, events must satisfy $S_{\mathrm {T}} <1200\,(\ge 1200)\,\text {Ge}\text {V}$. This division adds sensitivity for $\mathrm {LQ}_3$ masses of 600$\,\text {Ge}\text {V}$ and higher.

The top quarks originating from the decay of a heavy $\mathrm {LQ}_3$ are expected to be produced with larger $p_{\mathrm {T}}$ than the top quarks produced in background processes. Therefore, the transverse momentum distribution of the top quark candidate decaying into hadronic jets ($p_{\mathrm {T}}^{\mathrm {t}}$) gives discrimination power between background and signal events, and a measurement of the $p_{\mathrm {T}}^{\mathrm {t}}$ spectrum is performed in category A.

A kinematic reconstruction of the top quark candidate is performed by building top quark hypotheses using between one and five jets. Because of the presence of multiple hypotheses in each event, we choose the hypothesis in which the reconstructed top quark mass is closest to the value of 172.5$\,\text {Ge}\text {V}$.

The statistical evaluation in this category is performed through a template-based fit to the measured $p_{\mathrm {T}}^{\mathrm {t}}$ distribution.

### Category B: $\ell \tau _\mathrm {h} \tau _\mathrm {h}$ + jets 

In this category events are required to have at least two $\tau _\mathrm {h}$ leptons and one electron or muon. This requirement of two $\tau _\mathrm {h}$ leptons removes a large fraction of the SM background processes. The exception to this exclusion of SM backgrounds are diboson production events that contain one or more $\tau _\mathrm {h}$ leptons, but the cross sections for these processes are small. The selection criteria in this category are adapted to provide good sensitivity for low LQ masses.

Each event is required to contain an OS $\tau _\mathrm {h} \tau _\mathrm {h}$ pair. If the event contains more than one $\tau _\mathrm {h} \tau _\mathrm {h}$ pair, the OS pair with the largest scalar $p_{\mathrm {T}}$ sum is selected. Moreover, the leading and subleading $\tau _\mathrm {h}$ must satisfy $p_{\mathrm {T}} >65$ and $35\,\text {Ge}\text {V}$, respectively.

In this category a counting experiment is performed, as the number of expected background events is too small for results to benefit from a shape-based analysis.

## Background estimation

The background in this analysis consists of samples of events that are selected because of jets misidentified as $\tau _\mathrm {h}$ leptons and events with one electron or muon together with one or more $\tau _\mathrm {h}$ leptons.

In the following, events from ${\mathrm {t}\overline{\mathrm {t}}}$ and $\mathrm {W}+\text {jets}$ production that contain at least one misidentified $\tau _\mathrm {h}$ lepton are obtained from control regions (CRs) separately defined for the two search regions (SRs) A and B. We consider the following contributions: the ${\mathrm {t}\overline{\mathrm {t}}}$ background that consists of only misidentified $\tau _\mathrm {h}$ leptons (or exactly one misidentified $\tau _\mathrm {h}$ lepton as in category A), denoted by ${\mathrm {t}\overline{\mathrm {t}}} _{\mathrm {f}}$, the ${\mathrm {t}\overline{\mathrm {t}}}$ background that consists of (at least) one $\tau _\mathrm {h}$ lepton and (at least) one misidentified $\tau _\mathrm {h}$ lepton (only used in category B), denoted by ${\mathrm {t}\overline{\mathrm {t}}} _{\mathrm {p+f}}$, and the ${\mathrm {t}\overline{\mathrm {t}}}$ background that consists of one $\tau _\mathrm {h}$ lepton, denoted by ${\mathrm {t}\overline{\mathrm {t}}} _{\mathrm {p}}$.

An extrapolation method is used to derive the background due to misidentified $\tau _\mathrm {h}$ leptons. The normalization, and in category A also the shape, of the ${\mathrm {t}\overline{\mathrm {t}}}$ background is estimated using

1 $$\begin{aligned} N^{{\mathrm {t}\overline{\mathrm {t}}}, \, \text {data}}_{\text {SR}} = \left( N^{\text {data}}_{\text {CR}}-N^{\text {other, MC}}_{\text {CR}} \right) \, \frac{N^{{\mathrm {t}\overline{\mathrm {t}}}, \,\text {MC}}_{\text {SR}}}{N^{{\mathrm {t}\overline{\mathrm {t}}}, \, \text {MC}}_{\text {CR}}}, \end{aligned}$$

where *N* is the total number of events for the respective process in the signal region or control region and where “other” denotes all non-${\mathrm {t}\overline{\mathrm {t}}}$ background processes that are estimated from simulation. The contribution to the background from events with $\tau _\mathrm {h}$ leptons only is estimated from simulated events.

### Backgrounds in category A

In each subcategory of category A, the largest fraction of background events originates from ${\mathrm {t}\overline{\mathrm {t}}}$ production. The second largest source of background events arises from $\mathrm {W}+\text {jets}$ production, while minor contributions come from single top quark and $\mathrm {Z} +\text {jets}$ production.

The ${\mathrm {t}\overline{\mathrm {t}}} _{\mathrm {f}}$ background and the $\mathrm {W}+\text {jets}$ background that contain a misidentified $\tau _\mathrm {h}$ lepton are derived from a single control region ($\mathrm {CR}_{\mathrm {A}}$), which is defined through the same selection requirements as for the SR, but with an inverted isolation requirement for the $\tau _\mathrm {h}$ lepton.

**Fig. 2:**
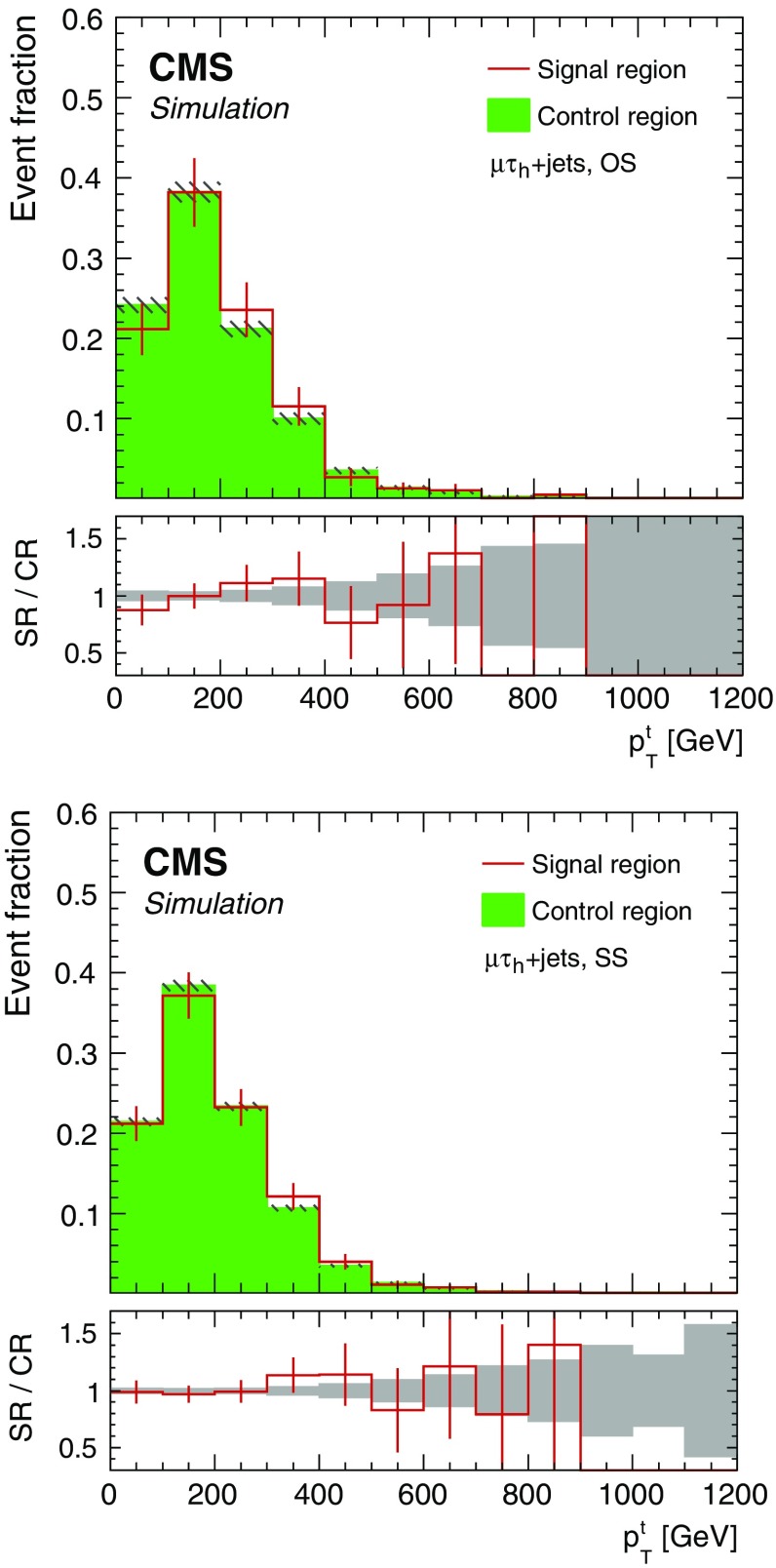
Shape comparison between the category A signal region and the corresponding control region, as a function of $p_{\mathrm {T}}^{\mathrm {t}}$, for simulated ${\mathrm {t}\overline{\mathrm {t}}}$ and $\mathrm {W}+\text {jets}$ events. Events with an opposite-sign $\mu \tau _\mathrm {h}$ pair are shown in the upper panel, while those with a same-sign $\mu \tau _\mathrm {h}$ pair are shown in the lower panel. The full selection is applied and the $S_{\mathrm {T}}$ categories are combined. All histograms are normalized to the total number of entries. Uncertainties of the signal region and control region are indicated by red error bars and gray hatched areas, respectively. The gray band in the ratio plot corresponds to the statistical uncertainty in the simulated samples

The shape of the $p_{\mathrm {T}}^{\mathrm {t}}$ distribution is compared between the $\mathrm {CR}_{\mathrm {A}}$ and SR in simulated ${\mathrm {t}\overline{\mathrm {t}}}$ and $\mathrm {W}+\text {jets}$ events. Since the inversion of the $\tau _\mathrm {h}$ isolation criterion introduces kinematic differences between the SRs and CRs, the jet multiplicity and $p_{\mathrm {T}}^{\mathrm {t}}$ are corrected in order to reproduce the shape of the ${\mathrm {t}\overline{\mathrm {t}}}$ and $\mathrm {W}+\text {jets}$ backgrounds in the SRs [71], as shown in Fig. 2.

Once the kinematic distributions in the $\mathrm {CR}_{\mathrm {A}}$ are corrected, we use Eq. (1) to extrapolate the ${\mathrm {t}\overline{\mathrm {t}}}$ and $\mathrm {W}+\text {jets}$ background yields to the SR. In this equation, we replace $N^{{\mathrm {t}\overline{\mathrm {t}}}}$ with $N^{{\mathrm {t}\overline{\mathrm {t}}},\,\mathrm {W}+\text {jets}}$ for category A.

### Backgrounds in category B

**Fig. 3:**
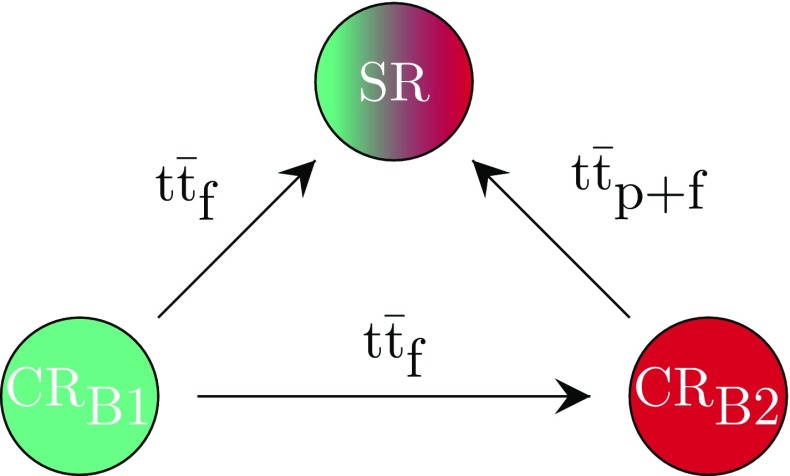
Strategy for the background estimation in category B. The ${\mathrm {t}\overline{\mathrm {t}}} _{\mathrm {f}}$ background in the signal region is derived from the control region $\mathrm {CR}_{\mathrm {B1}}$. The ${\mathrm {t}\overline{\mathrm {t}}} _{\mathrm {p+f}}$ background in the signal region is derived from the control region $\mathrm {CR}_{\mathrm {B2}}$. To obtain an estimate of the ${\mathrm {t}\overline{\mathrm {t}}} _{\mathrm {f}}$ background in the control region $\mathrm {CR}_{\mathrm {B2}}$, the control region $\mathrm {CR}_{\mathrm {B1}}$ is used

In category B, the dominant background also originates from ${\mathrm {t}\overline{\mathrm {t}}}$ production. As the fraction of misidentified electrons and muons was found to be negligible in this analysis, at least one of the two $\tau _\mathrm {h}$ leptons is mimicked by a jet. Thus, background events from ${\mathrm {t}\overline{\mathrm {t}}}$ production consist either of only misidentified $\tau _\mathrm {h}$ leptons or one $\tau _\mathrm {h}$ lepton and one misidentified $\tau _\mathrm {h}$ lepton, plus an electron or a muon. A separate CR is defined for each component. The strategy for determining this background in category B is shown in Fig. 3.

The first control region ($\mathrm {CR}_{\mathrm {B1}}$) is defined by inverting the isolation criterion for all $\tau _\mathrm {h}$ leptons with respect to the isolation criterion applied in the SR. The region $\mathrm {CR}_{\mathrm {B1}}$ is used to extrapolate the ${\mathrm {t}\overline{\mathrm {t}}} _{\mathrm {f}}$ background to the SR. In contrast to the SR, the charge criterion on the $\tau _\mathrm {h}$ lepton is removed and the leading $\tau _\mathrm {h}$ lepton must have $p_{\mathrm {T}} <100\,\text {Ge}\text {V}$ to avoid overlap between the control region $\mathrm {CR}_{\mathrm {B1}}$ and control region $\mathrm {CR}_{\mathrm {A}}$. The ${\mathrm {t}\overline{\mathrm {t}}} _{\mathrm {f}}$ background normalization is then derived as in Eq. (1).

A second control region ($\mathrm {CR}_{\mathrm {B2}}$) to estimate the ${\mathrm {t}\overline{\mathrm {t}}} _{\mathrm {p+f}}$ background is defined, in which at least one isolated and at least one nonisolated $\tau _\mathrm {h}$ lepton are required. In contrast to the SR, the charge criterion on the $\tau _\mathrm {h}$ lepton is removed and the leading $\tau _\mathrm {h}$ lepton must have $p_{\mathrm {T}} <45\,\text {Ge}\text {V}$. The event must have an opposite-sign $\ell \tau _\mathrm {h}$ pair. For this requirement, the pair with the largest summed $p_{\mathrm {T}}$ is chosen. In addition, the events must satisfy $M_{\mathrm {T}}(\ell ,p_{\mathrm {T}} ^\text {miss})>100\,\text {Ge}\text {V}$, where $M_{\mathrm {T}}(\ell ,p_{\mathrm {T}} ^\text {miss})$ is the transverse mass of the lepton-$\vec {p}_{\mathrm {T}}^{\,\text {miss}}$ system and defined as

$$\begin{aligned} \smash [b]{M_{\mathrm {T}}(\ell ,p_{\mathrm {T}} ^\text {miss})=\sqrt{2p_{\mathrm {T}} ^\ell p_{\mathrm {T}} ^\text {miss} \left( 1-\cos [\varDelta \phi (\vec {p}_{\mathrm {T}}^{\,\ell },\vec {p}_{\mathrm {T}}^{\,\text {miss}})]\right) }}. \end{aligned}$$

The largest non-${\mathrm {t}\overline{\mathrm {t}}} _{\mathrm {p+f}}$ fraction in control region $\mathrm {CR}_{\mathrm {B2}}$ arises from the ${\mathrm {t}\overline{\mathrm {t}}} _{\mathrm {f}}$ events. The estimate of this background is derived from the control region $\mathrm {CR}_{\mathrm {B1}}$ and extrapolated to the control region $\mathrm {CR}_{\mathrm {B2}}$ by using the extrapolation method as in Eq. (1). Once the ${\mathrm {t}\overline{\mathrm {t}}} _{\mathrm {f}}$ background is estimated from $\mathrm {CR}_{\mathrm {B1}}$, it is subtracted from $\mathrm {CR}_{\mathrm {B2}}$. The ${\mathrm {t}\overline{\mathrm {t}}} _{\mathrm {p+f}}$ background is extrapolated to the SR by using the extrapolation method as in Eq. (1).

## Systematic uncertainties

Systematic uncertainties can affect both the overall normalization of background components, and the shapes of the $p_{\mathrm {T}}^{\mathrm {t}}$ distributions for signal and background processes. Uncertainties in the MC simulation are applied to all simulated events used in the signal and in the various control regions. For each systematic uncertainty, the background estimation procedure described in Sect. 6 is repeated to study the impact of the respective systematic variation on the final result of the analysis. In the following, the systematic uncertainties applied to the analysis are summarized.

- The uncertainty in the integrated luminosity measurement recorded with the CMS detector in the 2016 run at $\sqrt{s}=13\,\text {Te}\text {V}$ is 2.5% [42].
- The following uncertainties in the normalization of the background processes are included:
  - 5.6% in the ${\mathrm {t}\overline{\mathrm {t}}}$ production cross sect. [72] for ${\mathrm {t}\overline{\mathrm {t}}}$ events that include $\tau$ leptons,
  - 10% for single top quark [73–75], W+jets, and Z+jets production [76],
  - 20% for diboson production [77–79].
- The estimation of pileup effects is based on the total inelastic cross section. This cross section is determined to be 69.2$\,\text {mb}$. The uncertainty is taken into account by varying the total inelastic cross section by 5% [80].
- Simulated events are corrected for lepton identification, trigger, and isolation efficiencies. The corresponding scale factors are applied as functions of $|\eta |$ and $p_{\mathrm {T}}$. The systematic uncertainties due to these corrections are taken into account by varying each scale factor within its uncertainty.
- The scale factors for the jet energy scale and the jet energy resolution are determined as functions of $|\eta |$ and $p_{\mathrm {T}}$ [66]. The effect of the uncertainties in these scale factors are considered by varying the scale factors within their uncertainties. These variations are propagated to the measurement of the $p_{\mathrm {T}} ^\text {miss}$.
- Scale factors for the $\mathrm {b}$ tagging efficiencies are applied. These scale factors are measured as a function of the jet $p_{\mathrm {T}}$ [68]. The corresponding uncertainty is taken into account by varying the scale factors within their uncertainties.

**Table 2:** Summary of largest systematic uncertainties for the ${\mathrm {t}\overline{\mathrm {t}}} _{\mathrm {f}}$ (and $\mathrm {W}+\text {jets}$) and ${\mathrm {t}\overline{\mathrm {t}}} _{\mathrm {p+f}}$ backgrounds derived from data, for the ${\mathrm {t}\overline{\mathrm {t}}} _{\mathrm {p}}$ background obtained from simulation and for a leptoquark signal with a mass of 700$\,\text {Ge}\text {V}$. Shown are the ranges of uncertainties, which are dependent on the search regions and the lepton channel type

Uncertainty	Category A			Category B		
	${\mathrm {t}\overline{\mathrm {t}}} _{\mathrm {p}}$	${\mathrm {t}\overline{\mathrm {t}}} _{\mathrm {f}} \,+\,\mathrm {W}+\text {jets}$	$\mathrm {LQ}_3$	${\mathrm {t}\overline{\mathrm {t}}} _{\mathrm {f}}$	${\mathrm {t}\overline{\mathrm {t}}} _{\mathrm {p+f}}$	$\mathrm {LQ}_3$
Scales ($\mu _{\mathrm {F}}$, $\mu _{\mathrm {R}}$) (%)	26–42	1–7	–	5–7	2–6	–
$\tau$ ID (%)	8–9	0–1	9–11	0	5–6	18–20
Bkg. estimate (%)	–	6–18	–	26–30	30–38	–

- Various uncertainties in the $\tau$ lepton reconstruction are considered. An uncertainty of 5% in the $\tau$ lepton identification is applied, with an additional uncertainty of $0.2\,p_{\mathrm {T}}/(1\,\text {Te}\text {V})$. An uncertainty of 3% in the $\tau$ lepton energy scale is taken into account, and an uncertainty in the charge misidentification rate of 2% is applied [70].
- Parton distribution functions from the NNPDF 3.0 set are used to generate simulated events for both background and signal samples. The uncertainties in the PDFs are determined according to the procedure described in Ref. [81]. The associated PDF uncertainties in the signal acceptance are estimated following the prescription for the LHC [81].
- We consider uncertainties in the renormalization ($\mu _{\mathrm {R}}$) and factorization ($\mu _{\mathrm {F}}$) scales by varying the respective scales, both simultaneously and independently, by factors between 0.5 and 2.
- We apply an uncertainty in the background estimation method by varying the extrapolation factors for background processes without $\tau$ leptons within their uncertainties. An additional uncertainty due to the correction factors used to reweight events in control region $\mathrm {CR}_{\mathrm {A}}$ is applied.

**Fig. 4:**
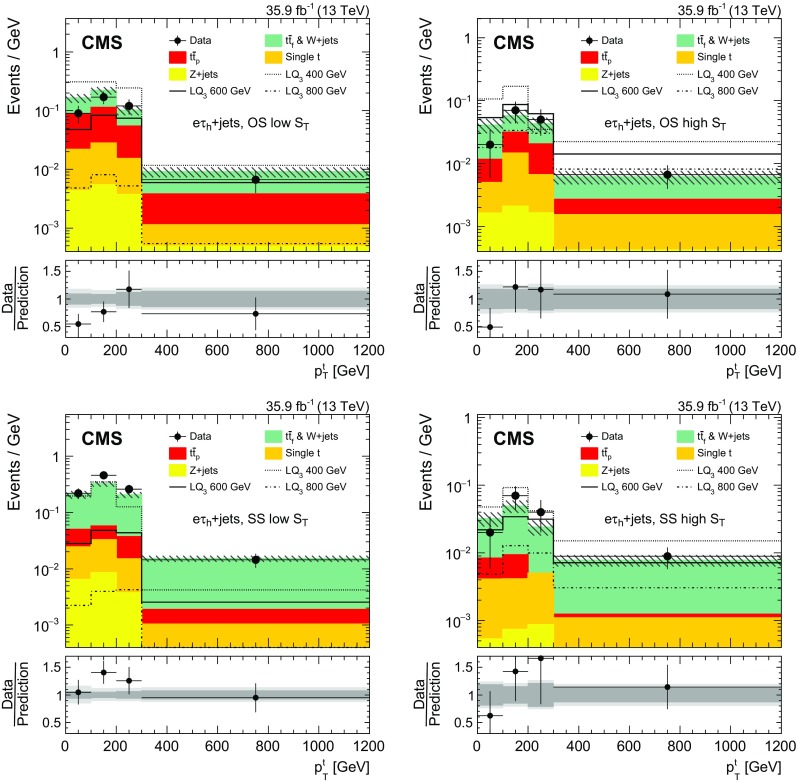
Distributions of $p_{\mathrm {T}}^{\mathrm {t}}$ for events in the electron channel passing the full selection in category A. The events are separated into OS (upper), SS (lower), low $S_{\mathrm {T}}$ (left) and high $S_{\mathrm {T}}$ (right) categories. The hatched areas represent the total uncertainties of the SM background. In the bottom panel, the ratio of data to SM background is shown together with statistical (dark gray) and total (light gray) uncertainties of the total SM background

The systematic uncertainties with the largest effects on the most important background processes and on the signal are summarized in Table 2. The most important background processes are the ${\mathrm {t}\overline{\mathrm {t}}} _{\mathrm {f}}$, ${\mathrm {t}\overline{\mathrm {t}}} _{\mathrm {f}}$ and $\mathrm {W}+\text {jets}$, and ${\mathrm {t}\overline{\mathrm {t}}} _{\mathrm {p+f}}$ backgrounds derived from data, and the ${\mathrm {t}\overline{\mathrm {t}}} _{\mathrm {p}}$ background taken from simulation. Also shown is the systematic uncertainty associated with the signal produced by an $\mathrm {LQ}_3$ whose mass is 700$\,\text {Ge}\text {V}$. The impact of the different sources of uncertainty varies for different processes. The uncertainty due to the variation in the scales $\mu _{\mathrm {R}}$ and $\mu _{\mathrm {F}}$ has a large impact on the ${\mathrm {t}\overline{\mathrm {t}}} _{\mathrm {p}}$ background, and is derived from simulation. The uncertainty in the $\tau$ lepton identification has the largest effect on the signal sample. For the backgrounds derived from several CRs, the uncertainty in the extrapolation factor has the largest impact.

## Results

The results of all search categories in the electron and muon channels are combined in a binned-likelihood fit. A statistical template-based analysis, using the measured $p_{\mathrm {T}}^{\mathrm {t}}$ distributions in category A and a counting experiment with the events measured in category B, is performed by using the Theta software package [82]. Each systematic uncertainty discussed in Sect. 7 is accounted for by a nuisance parameter in the likelihood formation.

**Fig. 5:**
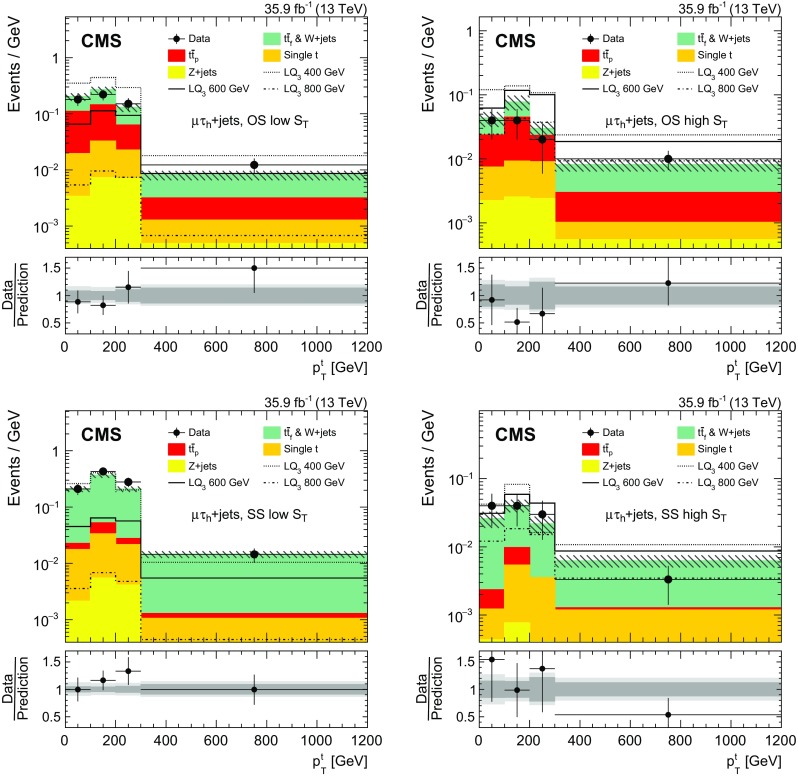
Distributions of $p_{\mathrm {T}}^{\mathrm {t}}$ for events in the muon channel passing the full selection in category A. The events are separated into OS (upper), SS (lower), low $S_{\mathrm {T}}$ (left) and high $S_{\mathrm {T}}$ (right) categories. The hatched areas represent the total uncertainties of the SM background. In the bottom panel, the ratio of data to SM background is shown together with statistical (dark gray) and total (light gray) uncertainties of the total SM background

The post-fit $p_{\mathrm {T}}^{\mathrm {t}}$ distributions in the electron and muon channels in category A are shown in Figs. 4 and  5, respectively. Contributions from ${\mathrm {t}\overline{\mathrm {t}}}$ and $\mathrm {W}+\text {jets}$ production with a misidentified $\tau _\mathrm {h}$ lepton are derived from control region $\mathrm {CR}_{\mathrm {A}}$, whereas SM backgrounds with a $\tau _\mathrm {h}$ lepton and other small backgrounds are taken from simulation.

**Table 3:** Final event yield in category B in the muon and electron channels for different leptoquark mass hypotheses, the background processes, and data. The total uncertainties for the signal and the background processes are shown

Process	$\mathrm {e}\tau _\mathrm {h} \tau _\mathrm {h} + \hbox {jets}$	$\mu \tau _\mathrm {h} \tau _\mathrm {h} + \hbox {jets}$
$\mathrm {LQ}_3$ (300$\,\text {Ge}\text {V}$)	97$^{+ 25 }_{- 24 }$	167$^{+ 36 }_{- 37 }$
$\mathrm {LQ}_3$ (400$\,\text {Ge}\text {V}$)	73$^{+ 14 }_{- 13 }$	98$^{+ 19 }_{- 17 }$
$\mathrm {LQ}_3$ (500$\,\text {Ge}\text {V}$)	34.1$^{+ 6.6 }_{- 6.2 }$	44.9$^{+ 8.5 }_{- 7.9 }$
$\mathrm {LQ}_3$ (600$\,\text {Ge}\text {V}$)	14.1$^{+ 2.8 }_{- 2.7 }$	21.1$^{+ 4.1 }_{- 3.8 }$
$\mathrm {LQ}_3$ (700$\,\text {Ge}\text {V}$)	7.3$^{+ 1.5 }_{- 1.4 }$	7.1$^{+ 1.5 }_{- 1.4 }$
$\mathrm {LQ}_3$ (800$\,\text {Ge}\text {V}$)	3.2$^{+ 0.7 }_{- 0.7 }$	4.4$^{+ 1.0 }_{- 0.9 }$
$\mathrm {LQ}_3$ (900$\,\text {Ge}\text {V}$)	1.5$^{+ 0.4 }_{- 0.3 }$	1.9$^{+ 0.4 }_{- 0.4 }$
$\mathrm {LQ}_3$ (1000$\,\text {Ge}\text {V}$)	0.8$^{+ 0.2 }_{- 0.2 }$	0.9$^{+ 0.2 }_{- 0.2 }$
$${\mathrm {t}\overline{\mathrm {t}}} _{\mathrm {f}}$$	2.5$$ ^{+ 0.8 }_{- 1.2 }$$	3.2$$ ^{+ 1.5 }_{- 1.2 }$$
$${\mathrm {t}\overline{\mathrm {t}}} _{\mathrm {p+f}}$$	1.5$$ ^{+ 0.8 }_{- 0.8 }$$	2.0$$ ^{+ 0.8 }_{- 0.9 }$$
Single t	0.3$$ ^{+ 0.3 }_{- 0.3 }$$	0.0$$ ^{+ 0.2 }_{- 0.0 }$$
W+jets	0.5$$ ^{+ 1.2 }_{- 0.5 }$$	0.4$$ ^{+ 0.7 }_{- 0.4 }$$
Z+jets	1.4$$ ^{+ 0.5 }_{- 0.5 }$$	1.0$$ ^{+ 0.4 }_{- 0.4 }$$
Diboson	1.6$$ ^{+ 1.7 }_{- 1.6 }$$	1.7$$ ^{+ 1.8 }_{- 1.7 }$$
Total background	7.9$$ ^{+ 2.4 }_{- 2.5 }$$	8.4$$ ^{+ 2.6 }_{- 2.3 }$$
Data	9	11

In Table 3, the total number of events from background processes and signal processes in category B is summarized. No significant deviation from the SM prediction is observed in the data in either category A or category B.

A Bayesian statistical method [82, 83] is used to derive 95% confidence level ($\text {CL}$) upper limits on the product of the cross section and the branching fraction squared for $\mathrm {LQ}_3$ pair production. Pseudo-experiments are performed to extract expected limits under a background-only hypothesis. For the signal cross section parameter, we use a uniform prior distribution. For the nuisance parameters, log-normal prior distributions are used. These are randomly varied within their ranges of validity to estimate the 68 and 95% $\text {CL}$ expected limits. Correlations between the systematic uncertainties across all channels are taken into account. The statistical uncertainties of simulated samples are treated as an additional Poisson nuisance parameter in each bin of the $p_{\mathrm {T}}^{\mathrm {t}}$ distribution.

**Fig. 6:**
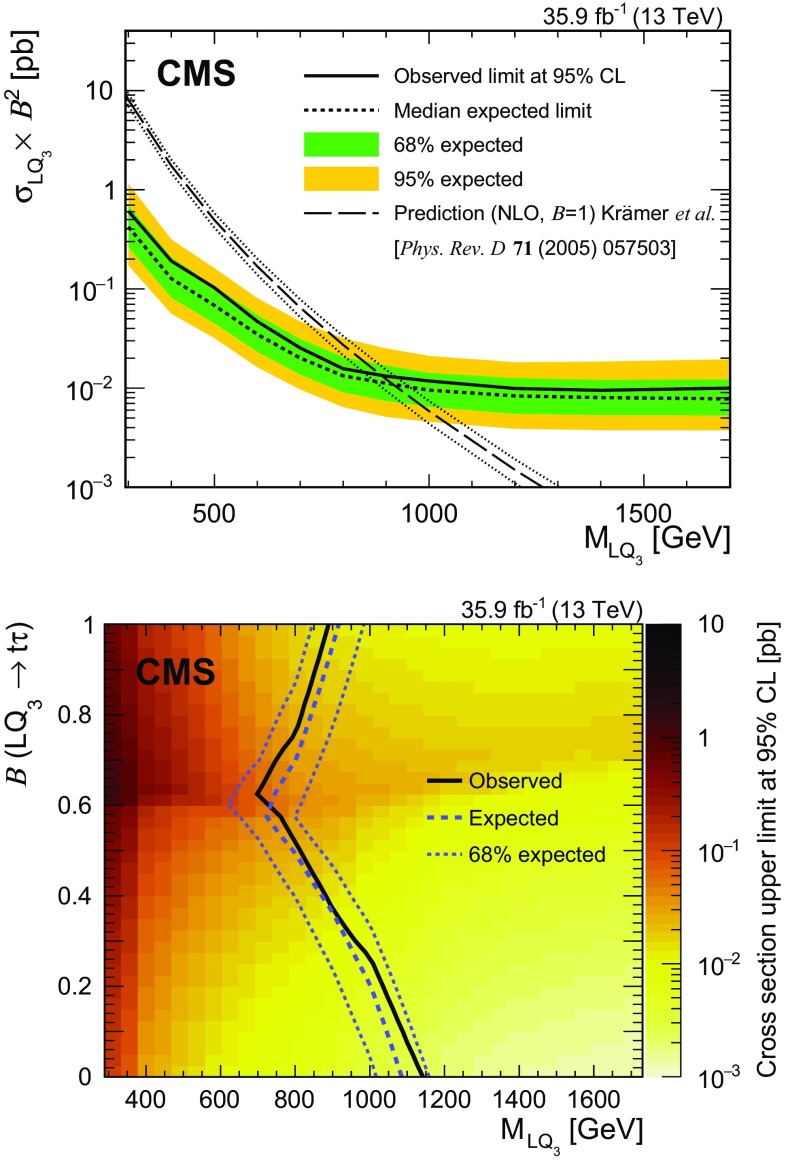
Upper limits at 95% confidence level on the product of the cross section and the branching fraction squared (upper), and on the leptoquark mass as a function of the branching fraction (lower), for the pair production of scalar LQs decaying to a top quark and a $\tau$ lepton. In the top plot, the theoretical curve corresponds to the NLO cross section with uncertainties from PDF and scale variations [24], shown by the dotted lines. The bottom plot additionally includes results from a search for pair-produced bottom squarks [38]

The 95% $\text {CL}$ upper limits on the product of the cross section and the branching fraction squared $\mathcal {B}^2$ as a function of $\mathrm {LQ}_3$ mass and the 95% $\text {CL}$ upper limits on the $\mathrm {LQ}_3$ mass as a function of $\mathcal {B}$ are shown in Fig. 6 (top). The cross section for pair production of scalar LQs at NLO accuracy [24] is shown as the dashed line. The dotted lines indicate the uncertainty due to the PDFs and to variations of the renormalization and factorization scales by factors of 0.5 and 2.

Production cross sections of 0.6$\,\text {pb}$ for $\mathrm {LQ}_3$ masses of 300$\,\text {Ge}\text {V}$ and of about 0.01$\,\text {pb}$ for masses up to 1.5$\,\text {Te}\text {V}$ are excluded at 95% $\text {CL}$ under the assumption of $\mathcal {B}=1$ for $\mathrm {LQ}_3$ decays to a top quark and $\tau$ lepton. Comparing these limits with the NLO cross sections, $\mathrm {LQ}_3$ masses up to 900$\,\text {Ge}\text {V}$ (930$\,\text {Ge}\text {V}$ expected) can be excluded.

Exclusion limits with varying branching fractions $\mathcal {B}$ are presented in Fig. 6 (bottom), where limits on the complementary $\mathrm {LQ}_3 \rightarrow \mathrm {b} \nu$ ($\mathcal {B}=0$) decay channel are also included. The results for $\mathcal {B}=0$ are obtained from a search for pair-produced bottom squarks [38] with subsequent decays into $\mathrm {b}$ quark and neutralino pairs, in the limit of vanishing neutralino masses. Scalar $\mathrm {LQ}_3$ s can be excluded for masses below 1150$\,\text {Ge}\text {V}$ for $\mathcal {B}=0$ and for masses below 700$\,\text {Ge}\text {V}$ over the full $\mathcal {B}$ range. For the assumptions of a LQ with symmetric couplings under the SM gauge symmetry and with decays to only $\mathrm {b} \nu$ and $\mathrm {t}\tau$, $\mathcal {B}$ can only take values of 1 or 0.5. When these assumptions are lifted, $\mathcal {B}$ can take all possible values between 0 and 1. Note that if upper limits on $\mathcal {B}$ are to be used to constrain the lepton-quark-$\mathrm {LQ}_3$ Yukawa couplings, $\lambda _{\mathrm {b} \nu }$ and $\lambda _{\mathrm {t} \tau }$, kinematic suppression factors that favor $\mathrm {b} \nu$ decay over the $\mathrm {t} \tau$ decay have to be considered as well [26, 27].

The results presented here can be directly reinterpreted in the context of pair produced down-type squarks decaying into top quark and $\tau$ lepton pairs. Such squarks appear in RPV SUSY scenarios and correspond to LQs with $\mathcal {B} = 0.5$. These squarks are excluded up to a mass of 810$\,\text {Ge}\text {V}$, and the decay mode is dominated by the RPV coupling $\lambda ^{\prime }_{333}$ [84].

## Summary

A search has been conducted for pair production of third-generation scalar leptoquarks ($\mathrm {LQ}_3$ s) decaying into a top quark and a $\tau$ lepton. Proton–proton collision data recorded in 2016 at a center-of-mass energy of 13$\,\text {Te}\text {V}$, corresponding to an integrated luminosity of 35.9$\,\text {fb}^{-1}$, has been analyzed. The search has been carried out in the $\ell \tau _\mathrm {h}$+jets and $\ell \tau _\mathrm {h} \tau _\mathrm {h}$+jets channels, where $\ell$ is either an electron or muon and $\tau _\mathrm {h}$ indicates a tau lepton decaying to hadrons. Standard model backgrounds due to misidentified $\tau _\mathrm {h}$ leptons are derived from control regions. The measured transverse momentum distributions for the reconstructed top quark candidate are analyzed in four search regions in the $\ell \tau _\mathrm {h}$+jets channel. The observed number of events are found to be in agreement with the background predictions.

Upper limits on the production cross section of $\mathrm {LQ}_3$ pairs are set between 0.6 and 0.01$\,\text {pb}$ at 95% confidence level for $\mathrm {LQ}_3$ masses between 300 and 1700$\,\text {Ge}\text {V}$, assuming a branching fraction of $\mathcal {B} = 1$. The scalar $\mathrm {LQ}_3$ s are excluded with masses below 900$\,\text {Ge}\text {V}$, for $\mathcal {B}=1$. This result represents the most stringent limits to date on $\mathrm {LQ}_3$ s coupled to $\tau$ leptons and top quarks and constrains models explaining flavor anomalies in the $\mathrm {b}$ quark sector through contributions from scalar LQs.
